# Genome-wide Association Mapping Identifies a New Arsenate Reductase Enzyme Critical for Limiting Arsenic Accumulation in Plants

**DOI:** 10.1371/journal.pbio.1002009

**Published:** 2014-12-02

**Authors:** Dai-Yin Chao, Yi Chen, Jiugeng Chen, Shulin Shi, Ziru Chen, Chengcheng Wang, John M. Danku, Fang-Jie Zhao, David E. Salt

**Affiliations:** 1 National Key Laboratory of Plant Molecular Genetics (NKLPMG), Institute of Plant Physiology and Ecology, Shanghai Institutes for Biological Sciences, Chinese Academy of Sciences, Shanghai, China; 2 Institute of Biological and Environmental Sciences, University of Aberdeen, Aberdeen, United Kingdom; 3 Sustainable Soils and Grassland Systems Department, Rothamsted Research, Harpenden, Hertfordshire, United Kingdom; 4 State Key Laboratory of Crop Genetics and Germplasm Enhancement, College of Resources and Environmental Sciences, Nanjing Agricultural University, Nanjing, China; 5 University of Chinese Academy of Sciences, Beijing, China; 6 Rothamsted Research, Harpenden, Hertfordshire, United Kingdom; University of California Davis, United States of America

## Abstract

Inorganic arsenic is a carcinogen, and its ingestion through foods such as rice presents a significant risk to human health. Plants chemically reduce arsenate to arsenite. Using genome-wide association (GWA) mapping of loci controlling natural variation in arsenic accumulation in *Arabidopsis thaliana* allowed us to identify the arsenate reductase required for this reduction, which we named High Arsenic Content 1 (HAC1). Complementation verified the identity of *HAC1*, and expression in *Escherichia coli* lacking a functional arsenate reductase confirmed the arsenate reductase activity of HAC1. The HAC1 protein accumulates in the epidermis, the outer cell layer of the root, and also in the pericycle cells surrounding the central vascular tissue. Plants lacking *HAC1* lose their ability to efflux arsenite from roots, leading to both increased transport of arsenic into the central vascular tissue and on into the shoot. HAC1 therefore functions to reduce arsenate to arsenite in the outer cell layer of the root, facilitating efflux of arsenic as arsenite back into the soil to limit both its accumulation in the root and transport to the shoot. Arsenate reduction by HAC1 in the pericycle may play a role in limiting arsenic loading into the xylem. Loss of *HAC1*-encoded arsenic reduction leads to a significant increase in arsenic accumulation in shoots, causing an increased sensitivity to arsenate toxicity. We also confirmed the previous observation that the ACR2 arsenate reductase in *A. thaliana* plays no detectable role in arsenic metabolism. Furthermore, *ACR2* does not interact epistatically with *HAC1*, since arsenic metabolism in the *acr2 hac1* double mutant is disrupted in an identical manner to that described for the *hac1* single mutant. Our identification of *HAC1* and its associated natural variation provides an important new resource for the development of low arsenic-containing food such as rice.

## Abstract 2

A genome-wide association study identifies the enzyme in plants that transforms arsenate into arsenite, allowing its extrusion into the soil and thereby controlling arsenic accumulation.

## Author Summary

Arsenic is a human carcinogen that accumulates from soil into many different food crops, where it presents a significantly increased cancer risk when foods derived from these crops are consumed. Plants naturally control the amount of arsenic they accumulate by first chemically converting arsenate into arsenite, which is then extruded from the roots back into the soil. Because arsenate is a chemical analogue of phosphate, conversion of arsenate in the root to arsenite may also prevent arsenic being efficiently transported to the shoots via the phosphate transport system. The chemical reduction of arsenate to generate arsenite is therefore clearly a key component of a plant's detoxification strategy. Here, we use genetic methods to identify the enzyme responsible for this crucial reaction—HAC1. We show that HAC1 is responsible for arsenate reductase activity in both the outer layer of the root (epidermis) and the inner layer adjacent to the xylem (pericycle). In its absence, the roots return less arsenic to the soil and the shoots accumulate up to 300 times more arsenic. This knowledge creates new opportunities to limit arsenic accumulation in food crops, thereby helping to reduce the cancer risk from this food-chain contaminant.

## Introduction

Inorganic arsenic is a non-threshold class-1 chronic exposure human carcinogen [1], and its elevated level in rice (*Oryza sativa*) produced in Bangladesh, China, and India is known to pose a significantly elevated cancer risk in these populations, which eat rice at the high levels typical of many Southeast Asian countries [2],[3]. Products derived from rice (such as baby food) and juices (such as apple and grape) can also contain inorganic arsenic at levels that pose a health risk. Several brands of baby food and juice contain arsenic concentrations that exceed the United States federal arsenic limit for drinking water [4],[5] raising significant health concerns in the US and Europe [6]–[8]. Because of this serious and widespread food safety concern research into understanding the mechanisms driving arsenic accumulation in plants has become a priority [9].

Arsenate is the most prevalent form of arsenic in the environment and its similarity to phosphate allows it to be taken up by plants via the phosphate uptake transporters [10]. On exposure to arsenate, plants rapidly respond by suppressing expression of the *PHT1;1* gene encoding an arsenate/phosphate transporter, and by removing the transport protein from the plasma membrane [11] to limit arsenate uptake. Expression of *PHT1;1* in response to arsenate is modulated by the transcription factor WRKY6 [11]. Though this response helps limit arsenate uptake, it does not eliminate it, and the first step after plants take up arsenate is its chemical reduction to arsenite [12]. In the arsenite form arsenic is either extruded back out of roots [13],[14], transported to the shoot (and on to the grain) [15],[16], or detoxified by complexation by thiol groups in phytochelatins and compartmentalised as a complex into the vacuole [12],[17]–[19]. The molecular components that drive these processes downstream of arsenate's conversion to arsenite are starting to be understood [20]. Using sequence homology with the known *Saccharomyces cerevisiae* arsenate reductase ACR2 [21], or functional complementation of a yeast mutant lacking a functional *ACR2*, genes encoding ACR2-like enzymes have been isolated from the plants *Arabidopsis thaliana*, rice, *Pteris vittata*, and *Holcus lanatus* [22]–[25]. Initial evidence using RNA interference (RNAi) suggested that suppression of the *ACR2*-like gene in *A. thaliana* caused a significant increase in sensitivity to arsenate and increased accumulation of arsenic [23]. However, more recent experiments using two independent T-DNA insertion alleles of the *ACR2*-like gene (*acr2-1* and *acr2*-2) showed that specific loss-of-function of this gene in *A. thaliana* has no observable impact on arsenate tolerance, the accumulation of arsenate or arsenite, or the efflux of arsenite from roots [26]. The function of this *ACR2*-like gene in arsenic metabolism in *A. thaliana* now appears unlikely.

Natural genetic variation is a powerful resource for investigating the molecular function of genes [27]. *A. thaliana* is broadly distributed throughout the northern hemisphere, and its genome contains extensive diversity associated with broad phenotypic variability [28] and local adaptation [29]–[34]. This natural variation has been used to identify specific genes involved in controlling variation in many traits [28]. Connecting natural genetic variation with its associated phenotype(s) has traditionally been achieved using populations of recombinant inbred lines (RILs) in which homozygous alternative alleles are segregating. Such populations have high sensitivity to detect causal loci. However, they have low resolving power because of the limited number of recombination events, making identification of causal genes more difficult. Furthermore, because each mapping population is usually generated from a cross between two parental accessions, only a limited amount of natural allelic diversity is captured in these populations, limiting the detection of important minor alleles. Genome-wide association (GWA) mapping is an alternative approach to using synthetic RILs, which takes advantage of the large number of historic recombination events within a population. By coupling these events with linked DNA polymorphisms genome-wide, the phenotypic effect of multiple alleles across the genome can be tested. However, unlike in synthetic RILs made from a bi-parental cross, the low frequency of rare alleles in a natural population makes it difficult to detect their phenotypic effect using GWA mapping. Nonetheless, GWA mapping has been successfully used in plants [35], including *A. thaliana* [36]–[46], rice [47]–[52], and maize [53]–[58], for the identification of quantitative trait loci (QTL) and candidate genes for various ecological and agricultural traits. Here, we report the use of GWA mapping to identify a major locus involved in controlling variation in arsenic accumulation in plants. This locus encodes an arsenate reductase enzyme that lacks the conserved active site of the canonical yeast ACR2 [21]. This arsenate reductase functions to chemically reduce arsenate to arsenite in the outer cell layer of the root to allow efflux of arsenite from the root back into the soil. The localisation of this enzyme in the pericycle also suggests it provides the arsenate reductase capacity to block the transport of arsenic to shoots as the easily transportable phosphate analogue arsenate by reducing arsenate to arsenite in the stele.

## Results

### Genome-wide Association Mapping

**Figure 1:**
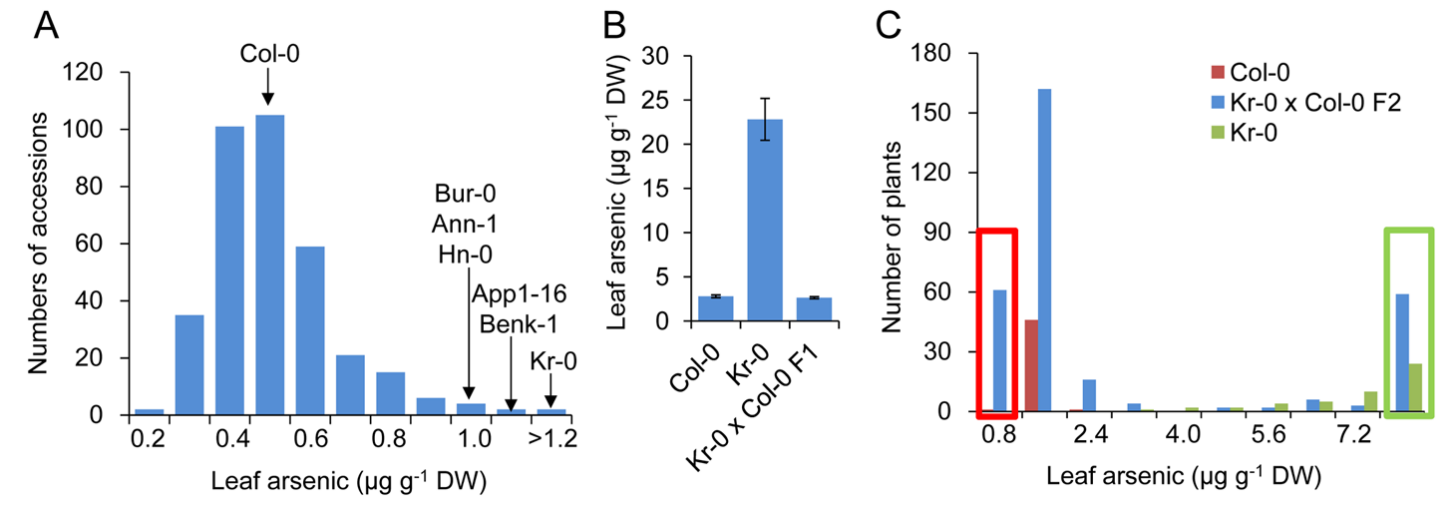
High leaf arsenic concentration in *A. thaliana* Kr-0 is controlled by a single recessive locus. (***A***) The frequency distribution of leaf arsenic concentrations in 349 *A. thaliana* accessions. Arrows indicate leaf arsenic concentration of accessions highlighted in the text. (***B***) Leaf arsenic concentration in *A. thaliana* accession Col-0, Kr-0, and their F1 progeny. Data represent the mean leaf arsenic concentration ± SE (*n* = 7–12). (***C***) The frequency distribution of leaf arsenic concentrations in F2 progeny of a cross between Kr-0 and Col-0. Red box indicates F2 plants used to create the low arsenic pool for XAM; Green box indicates F2 plants used to create the high arsenic pool for XAM. All leaf arsenic concentration data are accessible using the digital object identifiers (DOIs) 10.4231/T9H41PBV and 10.4231/T9QN64N6 (see http://dx.doi.org/) and available in Data S1.

**Figure 2:**
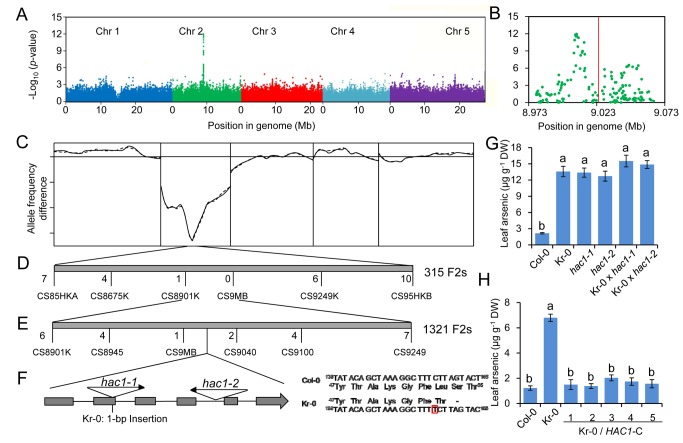
The *High Arsenic Content 1 (HAC1)* gene controls natural variation in leaf arsenic in *A. thaliana*. (***A***) Genome-wide association analysis of leaf arsenic concentration at 213,497 SNPs across 377 *A. thaliana* accessions using a mixed model analysis with correction for population structure. (***B***) A detailed plot of the peak region on chromosome 2 is shown with the location of *HAC1* indicated by the vertical red line. (***C***) DNA microarray-based bulk segregant analysis of the high leaf arsenic phenotype of Kr-0 using phenotyped F2 progeny from a Kr-0×Col-0 cross genotyped using the 256K AtSNPtilling microarray. Lines represent allele frequency differences between high and low leaf arsenic pools of F2 plants at SNPs known to be polymorphic between Kr-0 and Col-0 (Solid line = sense strand probes, dashed line = antisense strand probes). (***D***) The causal gene was mapped between CAPS makers CS8901K and CS9249K using 315 F2 plants. (***E***) Fine mapping narrowed *hac1* down to a 40 kb interval between markers CS9M and CS9040 using 1,321 F2 plants. Numbers below the horizontal line in (*D*) and (*E*) represent the number of recombinants between the indicated marker and *hac1*. (***F***) Gene structure of different *HAC1* alleles. Arrows indicate T-DNA insertion sites for *hac1-1* (GABI_868F11) *and hac1-2* (SM_3_38332). Grey boxes indicate exons, and black lines indicate introns. The causal polymorphism in Kr-0 is shown to the right. (***G***) Leaf arsenic concentrations of different *HAC1* alleles and their F1 progenies indicate through deficiency complementation that *HAC1* is the causal gene for the high leaf arsenic in Kr-0. (***H***) Kr-0 was transformed with the Col-0 genomic DNA fragment of *HAC1* (including 1.5Kb promoter sequence) and shown to complement the high leaf arsenic of Kr-0 to Col-0 levels in five independent transgenic lines (represented by numbers above the line in the x-axis legend), confirming *HAC1* is the causal gene for high leaf arsenic in Kr-0. Data in (*G*) and (*H*) represents the means ± S.E. (*n* = 4–12 independent plants per genotype). Letters above bars indicate statistically different groups using a one-way ANOVA followed by least significant difference (LSD) test at the probability of *p*<0.05. All leaf arsenic concentration data are accessible using the digital object identifiers (DOIs) 10.4231/T9H41PBV and 10.4231/T9VD6WCJ (see http://dx.doi.org/) and available in Data S2.

In this study we used 349 genetically diverse *A. thaliana* accessions collected from habitats across the species range [44],[46] to identify genetic loci controlling leaf accumulation of arsenic. To achieve this, plants were grown in artificial soil containing arsenate at a non-toxic, environmentally relevant, concentration of 0.1 µmoles g^−1^ dry weight (7.5 mg kg^−1^). After 5 wk of growth, and prior to flowering, one to two leaves were harvested from each plant and their total arsenic concentration determined by inductively coupled plasma–mass spectrometry (ICP-MS). Leaf arsenic concentrations were found to range from 0.15 to 3.49 µg g^−1^ dry weight (DW) (Figure 1A). To associate variation in leaf arsenic concentration with variation at specific genetic loci, we performed a GWA analysis. To achieve this, we used the leaf arsenic concentration in 337 of the phenotyped accessions that had previously been genotyped at approximately 248,584 genome-wide diallelic single nucleotide polymorphisms (SNPs) [46]. Applying a mixed model approach to associate genotype with phenotype at single loci while correcting for population structure [44],[46] revealed a 47 kb interval on chromosome 2 containing 30 SNPs that are highly associated with variation in leaf arsenic (*p*-values<10^−5^) (Figure 2A, 2B), 14 of which were associated with *p*-values<10^−8^. The most highly associated SNP (−log *p*-value = 11.9) at *Chr2:9008060* (TAIR9 genome release) explained 17.6% of the total variation in leaf arsenic concentration across the experiment. No SNPs in other chromosomal regions explaining more than 6% of the variation in leaf arsenic were observed, suggesting the causal gene in linkage with SNP *Chr2:9008060* is the major genetic locus controlling natural variation in leaf arsenic accumulation when *A. thaliana* is grown in soil containing a subtoxic concentration of arsenate. Accessions with a cytosine (C) at *Chr2:9008060* have average leaf arsenic concentrations 37.4% higher than accessions with a thymine (T), and the minor T allele is present in 24.3% of the 337 accessions studied.

### Genetic Validation of the Major Genome-wide Association

To validate the existence of this leaf arsenic QTL identified by GWA analysis we created a synthetic F2 recombinant population in which alternative alleles of the dialleic SNP *Chr2:9008060* were segregating. This population was created by crossing the high leaf arsenic accession Krefeld (Kr-0, CS28419), with a C at *Chr2:9008060*, to Col-0 that contains average leaf arsenic and has a T at *Chr2:9008060*. F1 plants from this cross had leaf arsenic concentrations equal to Col-0 (Figure 1B), indicating that the Kr-0 allele for high leaf arsenic is recessive relative to Col-0. Mapping the high arsenic locus was performed using extreme array mapping (XAM) [44],[59],[60]. Arsenic concentrations were measured in leaves from 315 F2 plants (Figure 1C) from the Kr-0×Col-0 cross and approximately one-quarter of these plants (75 of 315, X^2^ = 0.24<X^2^_0.05(1)_ = 3.84) had high leaf arsenic, suggesting that high leaf arsenic in Kr-0 is controlled by a single major locus. For XAM, 59 of these F2 plants with high leaf arsenic (As >7 µg g^−1^ DW) and 61 plants with low arsenic (As <0.8 µg g^−1^ DW) were pooled separately, genomic DNA isolated from each pool and genotyped using the Affymetrix SNP-tiling array Atsnptile 1. Allele frequency differences between the two pools for all SNPs polymorphic between Kr-0 and Col-0 were assessed [59] and used to determine that the causal locus for high leaf arsenic is located between 8.5 and 9.5 Mb on chromosome 2 (Figure 2C). This QTL was named *High Arsenic Content 1* (*HAC1*). Fine mapping based on the genotype and leaf arsenic concentrations of informative recombinants from a new set of 1,321 F2 plants from the Kr-0×Col-0 cross determined the causal locus for *HAC1* to be within a 40.9 kb region between markers CS9MB (SNP *Chr2:8993958*) and CS9040K (SNP *Chr2:9034914*) (Figure 2D, 2E).

### Identification of the *HAC1* Casual Gene

Linkage and GWA mapping located *HAC1* to the same region of chromosome 2 containing 18 genes (Figure 2A, 2E). To determine which gene is casual for *HAC1* we analysed the arsenic concentration of leaves from 48 T-DNA insertion alleles of genes in this region (Table S1). From this screen of T-DNA insertion alleles, we identified lines GABI_868F11 and SM_3_38332 with leaf arsenic concentrations similar to Kr-0, and we named them *hac1-1* and *hac1-2* (Figure 2F). Both mutants contained T-DNA insertions in gene At2g21045, suggesting this gene is causal for *HAC1*. The distance between the peak SNP from the GWA analysis and At2g21045 is 19.8 kb. Sequencing of At2g21045 revealed three polymorphic sites between Kr-0 and Col-0. Two of these are SNPs: one in an intron, and the other a synonymous SNP in an exon (Table S2). The other polymorphism is a 1-bp insertion in the second exon in Kr-0 causing replacement of Leu^53^ with a Thr and introducing a premature stop codon (Figure 2F) that truncates the protein by 116 amino acids. This truncation likely results in loss-of-function making this 1-bp insertion in At2g21045 responsible for high leaf arsenic in Kr-0. Loss-of-function alleles of *HAC1* were unable to complement the high leaf arsenic of Kr-0 in F1 hybrids (Figure 2G), whereas the Col-0 At2g21045 genomic fragment introduced transgenically into Kr-0 was able to complement (Figure 2H), confirming that At2g21045 is the causal gene for *HAC1*. At2g21045 was named *HAC1*. An analysis of *HAC1* in 220 re-sequenced *A. thaliana* accessions, and the Sanger sequencing of *HAC1* in five accessions with high leaf arsenic (App1-16, Benk-1, Bur-0, Ann-1, Hn-0) identified from our screen of 349 accessions, did not identify the 1 bp insertion observed in Kr-0 in any other accessions. This suggests that there is still significant functional allelic diversity at *HAC1* contributing to variation in leaf arsenic that needs to be described.

### HAC1 Functions As an Arsenate Reductase

**Figure 3:**
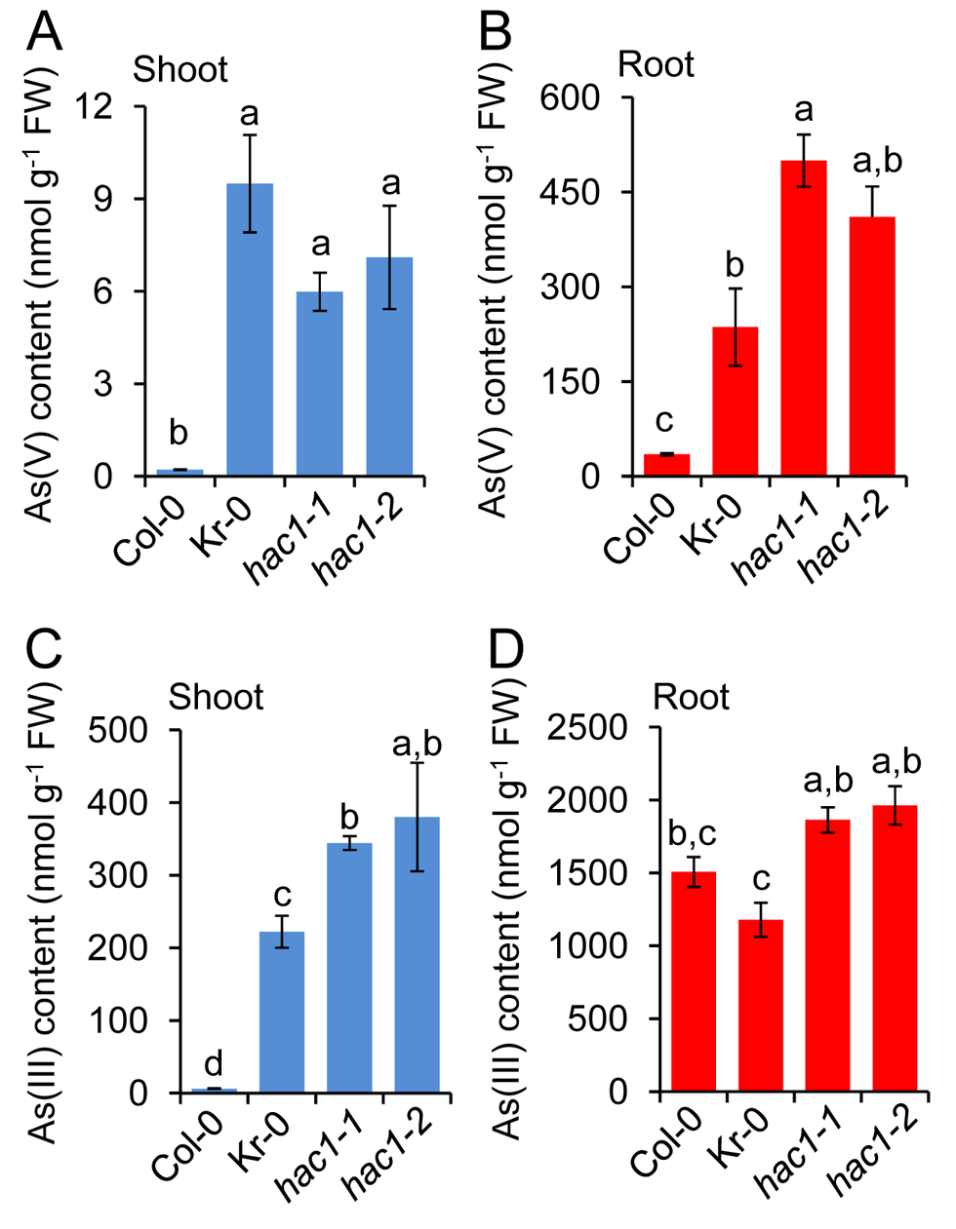
*HAC1* plays a central role in limiting arsenic accumulation during arsenate exposure. When grown in hydroponic media containing 5 µM arsenate, both Kr-0 and the two *hac1* null alleles show a clear increase in arsenate accumulation in shoots (***A***) and roots (***B***), and arsenite accumulation in shoots (***C***) but not in roots (***D***). Letters above bars indicate statistically different groups using a one-way ANOVA followed by least significant difference (LSD) test at the probability of *p*<0.05. Data represent means ± S.E. (*n* = 4). Raw data available in Data S3.

**Figure 4:**
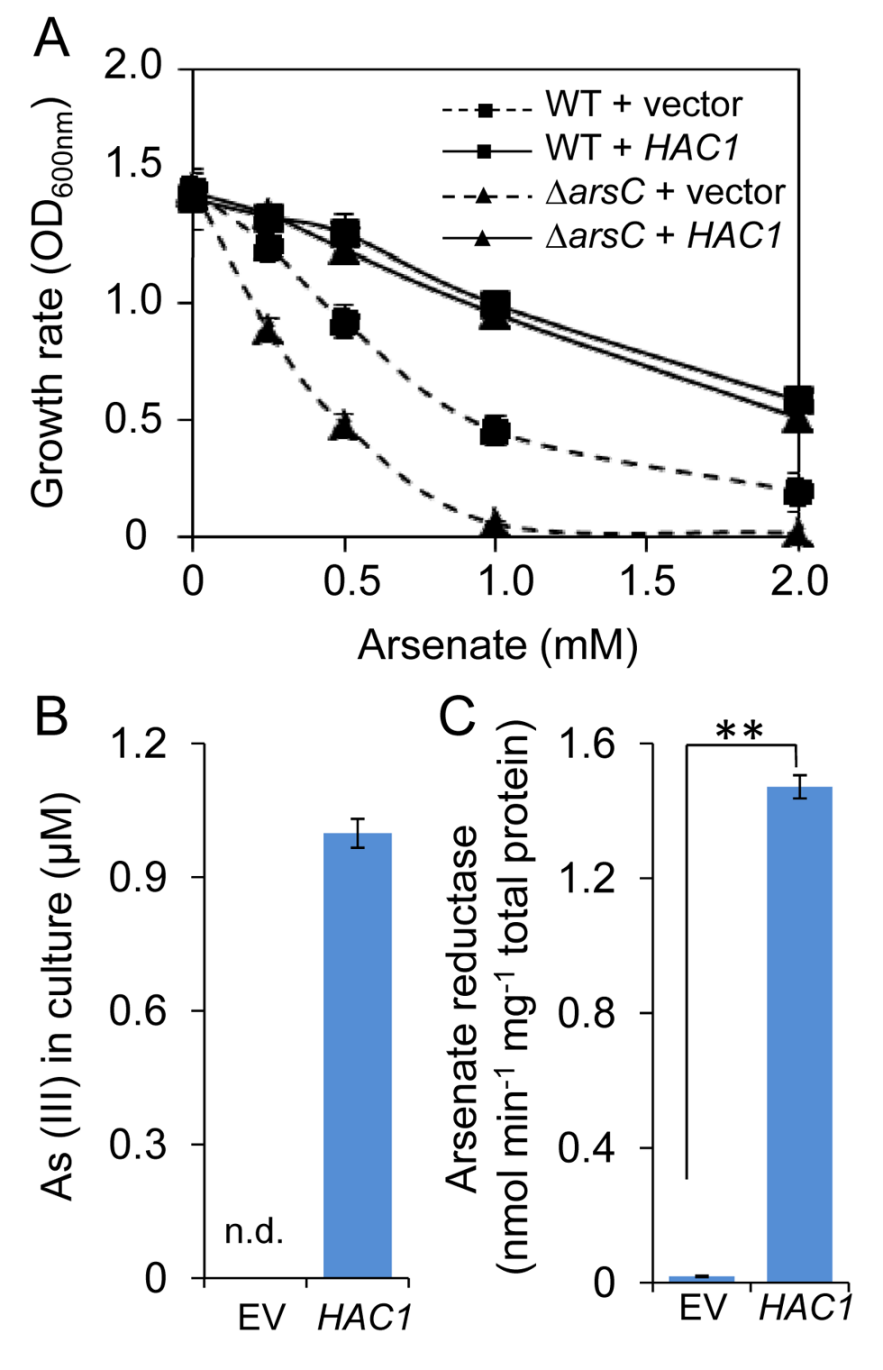
*HAC1* encodes an arsenate reductase. *HAC1* from *A. thaliana* suppresses the arsenate sensitivity of *E. coli* lacking the ArsC arsenate reductase. Strains were grown at 16°C and cell density measured at an optical density of 600 nm after 72 hr growth in different concentrations of arsenate (***A***). WT = *E. coli* wild type (W3110); *ΔarsC = arsC* mutant in WC3110; Vector = empty pCold-TF; *HAC1* = pCold-TF vector containing the *A. thaliana HAC1* gene (pCold-TF-*HAC1*). (***B***) After growth of *ΔarsC* transformed with pCold-TF-*HAC1* in media containing 10 µM arsenate for 72 hr arsenite was detected in the culture solution. However, arsenite was not detected after growth of *ΔarsC* transformed with pCold-TF empty vector. EV = empty pCold-TF; n.d = not detected. (***C***) Arsenate reductase activity in cell free extracts of *E. coli ΔarsC* mutant transformed with pCold-TF (EV) or pCold-TF-*HAC1* (*HAC1*). Arsenate reductase activity estimated as the oxidation of NADPH followed by a loss of absorbance at 340 nm. Data represents means ± S.E. (*n* = 3). Asterisks above bars in (**C**) represent a statistically significant difference (*p*<0.01), calculated using a Student's *t*-test. Raw data available in Data S4.

**Figure 5:**
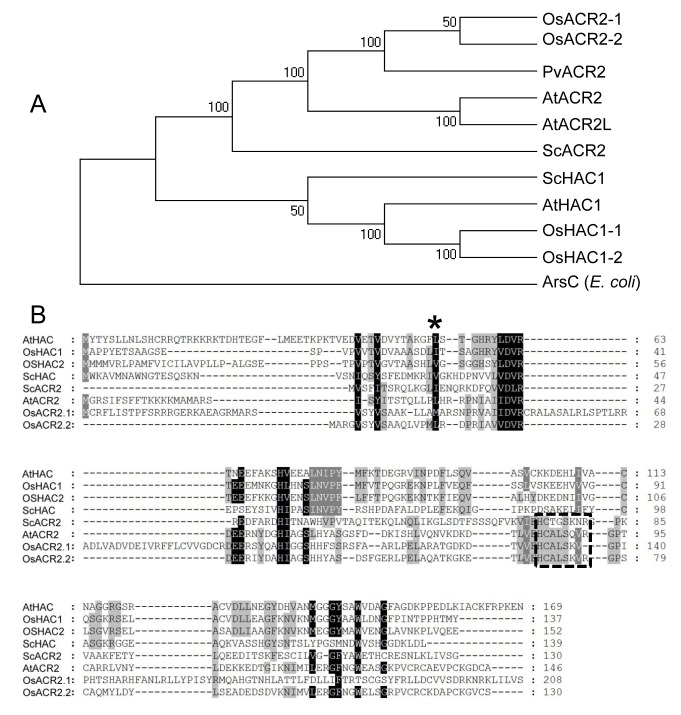
Sequence analysis of *HAC* and *ACR2* genes from plants and yeast. (***A***) A dendrogram showing the relationship among genes encoding arsenate reductase in *A. thaliana*, rice, yeast, and *E. coli*. Numbers at nodes show bootstrap values obtained from 1,000 replicate analyses. (***B***) Sequence alignment of *ACR2* and *HAC* genes. Asterisk represents the Leu^53^ converted into a Thr and followed by a stop codon in the *HAC1* Kr-0 allele. Dashed box represents the conserved catalytic site in the ACR2-like arsenate reductases. Gene codes for sequences used to generate the dendrogram are as follows; *OsHAC1-1* LOC_Os02g01220; *OsHAC1-2* LOC_Os04g17660; *OsACR2-1* LOC_Os10g39860; *OsACR2-2* LOC_Os03g01770; *AtACR2* AT5G03455; *ScHAC1* YOR285W; *PvACR2* DQ310370; *arsC* YP_005275964.

We observed that the predicted amino acid sequence encoded by *HAC1* contains a Rhodanase-like domain (IPR001763), a domain known to exist in arsenate reductase enzymes [61], in yeast [21], and in plants [22],[24],[25]. This suggested the possibility that *HAC1* functions as an arsenate reductase, reducing arsenate (As^V^) to arsenite (As^III^). To test this hypothesis, we evaluated the oxidation state of arsenic in plants lacking a functional *HAC1* gene. Consistent with *HAC1* encoding an arsenate reductase, both *hac1-1* and *hac1-2* loss-of-function alleles have increased accumulation of arsenate in shoots and roots compared to wild-type Col-0 (Figure 3A, 3B). Kr-0 also has increased arsenate accumulation in both shoots and roots, consistent with *HAC1^Kr-0^* being a loss-of-function allele. Moreover, in roots of *hac1-1*, *hac1-2*, and Kr-0 we observe a reduction in the percentage of the total accumulated arsenic present as arsenite. In roots of Col-0 wild-type plants 98% of the total accumulated arsenic is present as arsenite, whereas in roots of plants with a loss-of-function allele of *HAC1* (*hac1-1*, *hac1-2*, and Kr-0) arsenite accounts for between 79%–83% of total root arsenic. This reduced capacity to convert arsenate to arsenite is also associated with a significant increase in arsenic, primarily as arsenite, in the shoots (Figure 3C). We propose this increase in arsenic in shoots is driven by the enhanced accumulation of arsenate in the roots (Figure 3B), which is then readily translocated to the shoot. Once in the shoot this arsenate is reduced to arsenite via *HAC1*-independent mechanisms. As would be expected for an arsenate reductase, loss-of-function of *HAC1* had no effect on the accumulation or oxidation state of arsenite when plants were exposed to arsenite in the growth medium (Figure S1A, S1B). To further test the hypothesis that *HAC1* encodes an arsenate reductase, we heterologous expressed *HAC1^Col-0^* in a strain of *Escherichia coli* lacking its endogenous ArsC arsenate reductase. *E. coli* lacking ArsC is known to have enhanced sensitivity to arsenate toxicity because, without ArsC, arsenic is unable to be extruded from cells as arsenite, causing an accumulation of cellular arsenic that leads to enhanced arsenate sensitivity. We observed that heterologous expression of *HAC1* in the *ΔarsC* mutant lacking a functional copy of *arsC* suppressed this enhanced sensitivity to arsenate (Figure 4A), in a similar manner to other known plant and yeast arsenate reductases [23],[24],[62]. This suppression of arsenate sensitivity by *HAC1^Col-0^* was also associated with a recovery of the ability of the *ΔarsC E. coli* mutant to efflux arsenite back into the growth medium (Figure 4B). As expected, arsenate reductase activity is almost undetectable in a cell free extract of the *E. coli ΔarsC* mutant lacking an arsenate reductase (Figure 4C). However, heterologous expression of the *A. thaliana HAC1^Col-0^* gene in the *E. coli ΔarsC* loss-of-function mutant confers the ability to reduce arsenate to arsenite (Figure 4C). In this assay, arsenate reductase activity is monitored as the loss of the primary electron donor NADPH through its conversion into NADP^+^ during the coupled reduction of arsenate to arsenite. Plants are known to contain enzymes with arsenate reductase activity when tested in heterologous systems [22],[24],[25], and that shows sequence homology to the yeast arsenate reductase ACR2 (Figure 5A). These previously characterised plant enzymes contain the conserved HC*X*_5_R active site [63] found in the yeast ACR2 (Figure 5B), but these plant enzymes appear not to impact arsenic metabolism [26]. In contrast, *HAC1* in *A. thaliana* and its homologs in rice do not contain the yeast ACR2 canonical arsenate reductase active site (Figure 5B). HAC1 is well conserved in plants (Figure S2), with several domains of the protein having very high levels of conservation. However, further work is required to understand the functional significance of these highly conserved domains.

### HAC1 Functions in the Root to Limit Arsenic Accumulation

**Figure 6:**
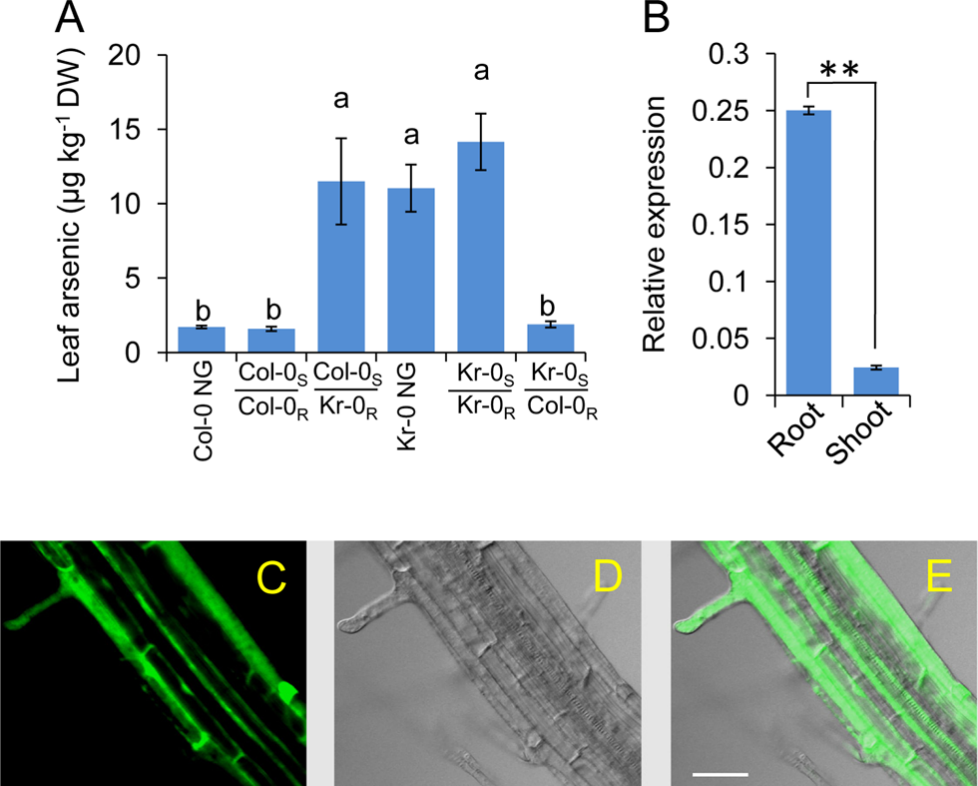
*HAC1* functions in the roots to limit arsenic accumulation in shoots. (***A***) Reciprocal grafting determines that the high leaf arsenic phenotype of Kr-0 is driven by the root. Col-0 NG = non-grafted Col-0; Kr-0 NG = non-grafted Kr-0; Col-0_S_/Col-0_R_ = Col-0 self grafted; Kr-0_S_/Kr-0_R_ = Kr-0 self grafted; Kr-0_S_/Col-0_R_ = Kr-0 shoot grafted onto a Col-0 root; Col-0_S_/Kr-0_R_ = Col-0 shoot grafted onto a Kr-0 root. (***B***) Quantitative real-time RT-PCR indicates *HAC1* is predominantly expressed in *A. thaliana* roots. Expression of *HAC1* was calculated as 2^−ΔCT^ relative to *UBC* (At5g25760). (***C*** **–** ***E***) Root specific expression of *HAC1* revealed by accumulation of the HAC1-GFP fusion protein driven by expression of *HAC1-GFP* by the *HAC1* native promoter in Col-0 wild type, imaged using a confocal microscope showing GFP fluorescence (***C***), bright light (***D***), and an overlay (***E***). Scale bar = 50 µm. Letters above bars in (***A***) indicate statistically different using a one-way ANOVA followed by least significant difference (LSD) test at the probability of *p*<0.05. Asterisks above bars in (***B***) represent a significant difference (*p*<0.01) using a Student's *t*-test. Data (***A*** and ***B***) represent means ± S.E. (*n* = 7–13 [***A***] and n = 4 [***B***]). Raw data available in Data S5.

**Figure 7:**
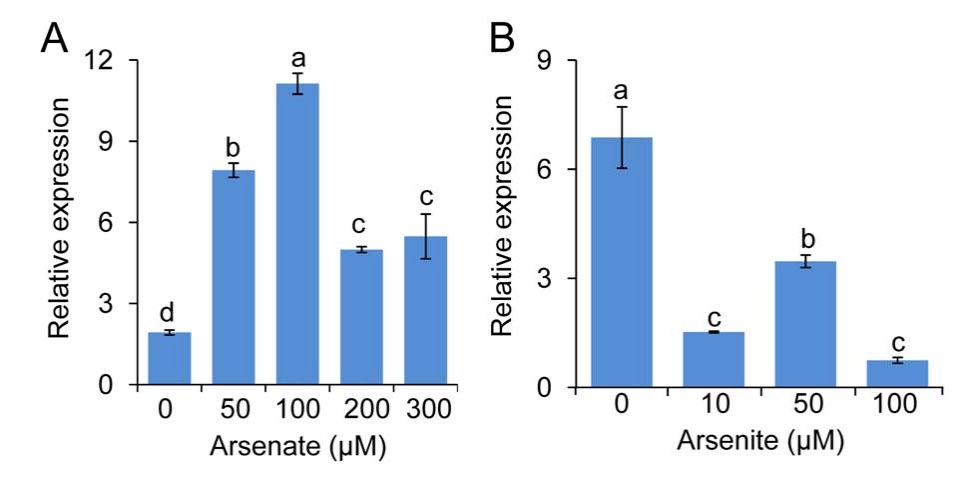
*HAC1* in roots is both constitutively expressed and induced by arsenate. Wild-type Col-0 plants were grown on agar solidified medium and, seven days after germination, were transferred to agar solidified medium containing various concentrations of arsenate (***A***) or arsenite (***B***), and after an additional three days, roots were harvested and *HAC1* expression level determined using quantitative real-time RT-PCR. Exposure to arsenate increased the steady state levels of *HAC1* mRNA in roots above the untreated control at all arsenate concentrations tested. However, similar treatment with arsenite reduced the steady state levels of *HAC1* mRNA in roots (***B***) at all concentrations tested. Letters above bars indicate statistically different groups using a one-way ANOVA followed by least significant difference (LSD) test at the probability of *p*<0.05. Data represent means ± S.E. (*n* = 3). Raw data available in Data S6.

We used reciprocal grafting to determine that a functional *HAC1* is required in roots to maintain a low arsenic concentration in shoots. Only plants with wild-type Col-0 roots containing a functional *HAC1* allele showed Col-0 like shoot arsenic concentrations, even when Col-0 roots are grafted onto Kr-0 shoots that contain a non-functional *HAC1* allele (Figure 6A). This root function of *HAC1* is consistent with *HAC1* being primarily expressed in roots (Figure 6B). Furthermore, transformation of Col-0 wild type with a *HAC1^Col-0^-Green Fluorescent Protein* (*HAC1^Col-0^-GFP*) construct expressed from the *HAC1* promoter allowed the detection of the HAC1-GFP fusion protein, which we observed to be localised to root hairs and epidermal cells at the surface of the root and to the pericycle within the stele (Figure 6C, 6E and Figure S3). Exposure to arsenate in agar solidified growth medium for 3 days causes a significant, dose dependent, increase in the steady state levels of *HAC1* mRNA in roots of Col-0 wild type (Figure 7A). This increase in *HAC1* mRNA levels reaches a maximum at 100 µM arsenate in the growth media (Figure 7A). In contrast, exposure to arsenite under the same conditions significantly reduces *HAC1* mRNA levels (Figure 7B) at all concentrations tested.

**Figure 8:**
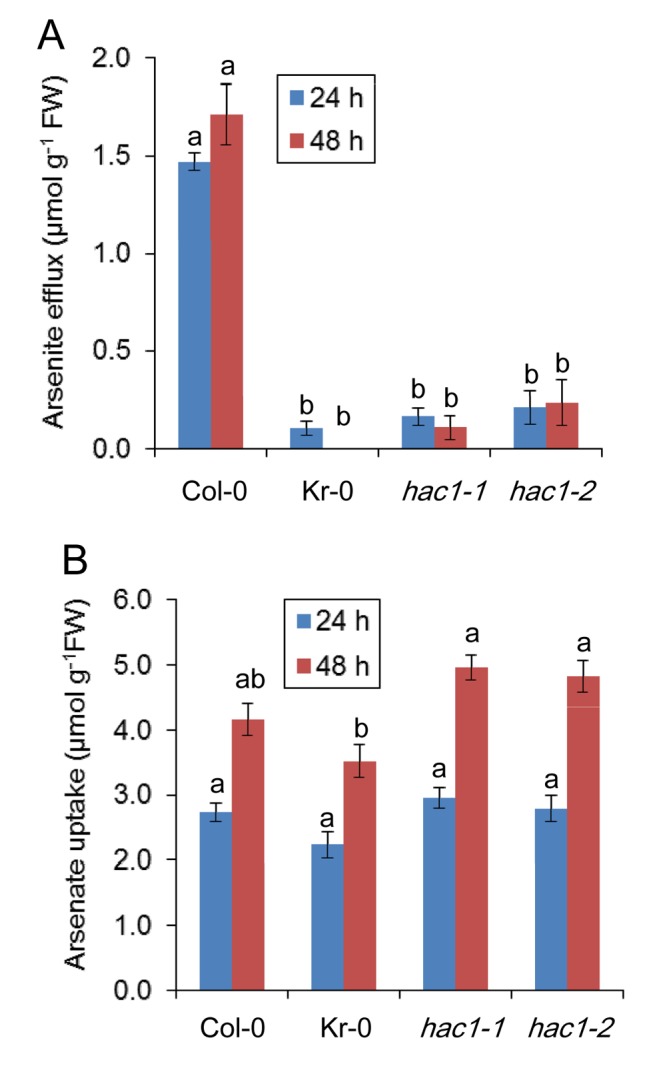
*HAC1* plays a central role in arsenic efflux. Both Kr-0 and the two *hac1* null alleles show a clear reduction in efflux of arsenite from roots compared to Col-0 after 24 and 48 hr exposure to arsenate in the hydroponic nutrient solution (***A***), whereas there is no difference in arsenate uptake from the same solution (***B***). All lines were grown hydroponically for 3 wk, and 5 µM arsenate were added for analysis thereafter. The uptake of arsenate and efflux of arsenite was calculated from changes in their concentration in the hydroponic growth media. Letters above bars indicate statistically different groups using a one-way ANOVA followed by least significant difference (LSD) test at the probability of *p*<0.05. Data represent means ± S.E. (*n* = 4). Raw data available in Data S7.

**Figure 9:**
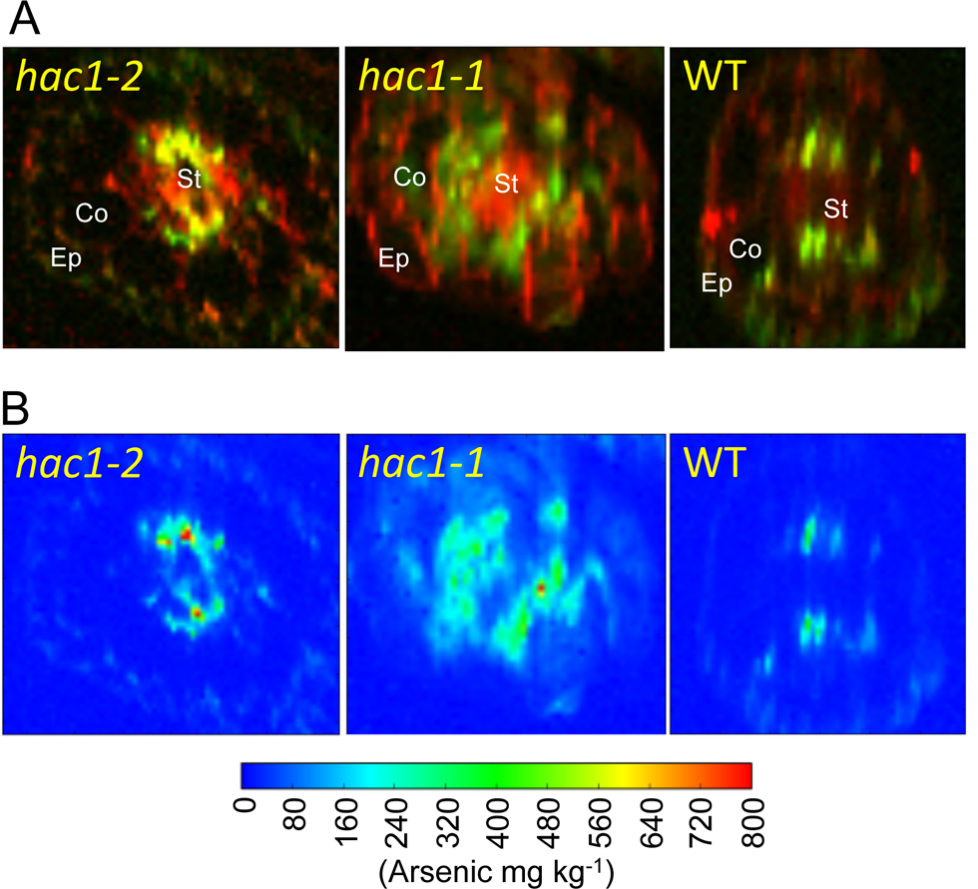
*HAC1* is required to limit arsenic accumulation in the stele. Synchrotron μ-XRF mapping of arsenic in root cross sections. Plants were exposed to 10 µM arsenate for 10 days in hydroponic solution. Root sections at approximately 2 cm from the tip were cut and prepared with high pressure freezing and freeze substitution and sectioned at 7 µm thickness. μ-XRF was performed at the UK Diamond Light Source with a beam size and step size = 2 µm and X-ray fluorescence detected using a silicon drift detector. (***A***) Both calcium (red) and arsenic (green) are imaged in wild-type Col-0 and both *hac1* mutant alleles to allow the localization of arsenic to be observed in relation to the overall cellular structure of the root marked by calcium within the cell walls. (***B***) Quantification of arsenic accumulation across the root section in the same samples shown in (***A***). Ep, epidermis; Co, cortex: St, stele.

To further understand the physiological role of HAC1 catalysed arsenate reduction in roots, we analysed both arsenite efflux and arsenate uptake by roots in *hac1* loss-of-function mutants and Kr-0 (Figure 8). To avoid artefacts caused by arsenic toxicity, these experiments were performed at 5 µM arsenate in the hydroponic nutrient solution, a subtoxic concentration of arsenate. We observed that arsenite efflux from roots, measured as the accumulation of arsenite in the nutrient media after exposure of the plants to arsenate, was dramatically reduced in both the *HAC1* loss-of-function mutants and Kr-0 compared to Col-0 wild-type plants (Figure 8A). Col-0 wild-type plants extruded 2 µmoles arsenite g^−1^ fresh weight of roots over a 48 hr period after exposure to arsenate. In contrast, in both *hac1-1* and *hac1-2* this efflux of arsenite was reduced 10-fold and was also reduced to a similar level in Kr-0, which also contains a loss-of-function allele of *HAC1*. On the other hand, arsenate uptake, measured as depletion of arsenate from the nutrient solution, was no different in *hac1-1*, *hac1-2*, or Kr-0 compared to wild-type Col-0 at both time points tested (Figure 8B). Such evidence establishes that *HAC1* is essential for arsenite efflux from roots, but is not necessary for arsenate uptake. To understand the role of HAC1-facilitated arsenite efflux in limiting arsenic translocation to the shoot, we investigated the distribution of arsenic in roots in the *hac1-1* and *hac1-2* loss-of-function mutants. Using Synchrotron μX-ray Fluorescence (μ-XRF) we were able to image arsenic accumulation at µm resolution in high pressure frozen, freeze-substituted sections of roots. Such imaging revealed that in the absence of a functional *HAC1* gene, and with the loss of arsenite efflux, arsenic appears to accumulate mainly in the root stele to concentrations 120%–180% higher than in the Col-0 wild type (Figure 9). The arsenic concentration in the scanned root sections of wild-type, *hac1-1* and *hac1-2* (Figure 9B) reached a maximum of 411, 917, and 1,154 mg kg^−1^, respectively.

### HAC1 Is Required for Arsenate Resistance

**Figure 10:**
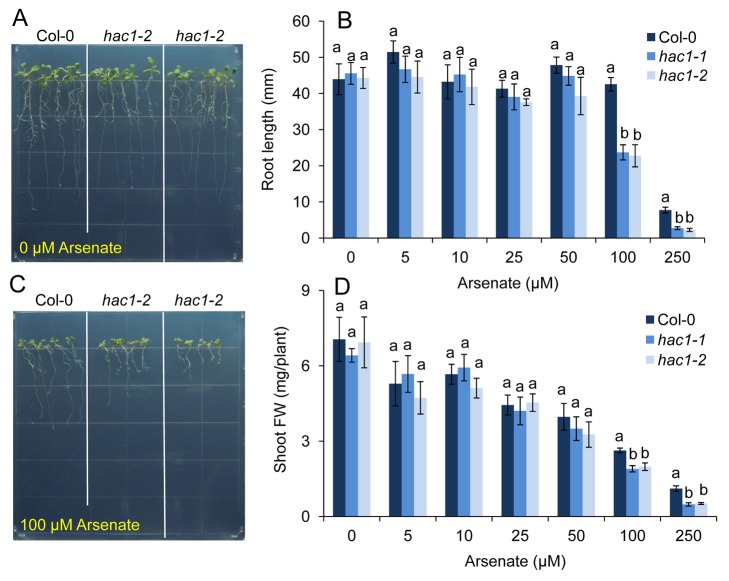
Loss-of-function of *HAC1* confers increased sensitivity to arsenate. Both Col-0 wild-type and the two *hac1* null alleles were grown in agar solidified nutrient medium containing 0 µM (***A***) and 100 µM (***C***) arsenate, and after 12 days a representative photograph taken. Plants were also grown in the same conditions on nutrient medium containing a range of arsenate concentrations and the root length (***B***) and shoot fresh weight (***D***) determined after 12 days of growth. Letters above bars in (***B***, ***D***) indicate statistically different groups within treatments using a one-way ANOVA followed by least significant difference (LSD) test at the probability of *p*<0.05. Data represent means ± S.E. (*n* = 4). Raw data available in Data S8.

We demonstrate that HAC1 plays a critical role in controlling the amount of arsenic accumulated in roots and shoots in *A. thaliana*. To evaluate if such a role is important in arsenic resistance, we exposed both *hac1-1* and *hac1-2* loss-of-function mutants to increasing concentrations of arsenate or arsenite in the agar solidified growth medium. Exposure to either arsenate (Figure 10) or arsenite (Figure S4) caused a significant reduction in both root growth and overall plant fresh weight (Figure 10 and Figure S4) for all genotypes. However, *hac1-1* and *hac1-2* were both significantly more sensitive to arsenate compared to the Col-0 wild type at relatively high concentrations of arsenate when growth was measured as root length or overall plant fresh weight (Figure 10). However, we observed no significant difference in sensitivity to arsenite between *hac1-1*, *hac1-2*, and Col-0 wild type (Figure S4). This observation supports our conclusion that HAC1 acts as an arsenate reductase in roots that controls arsenic accumulation in both roots and shoots when plants are exposed to arsenate.

### HAC1 Does Not Interact Epistatically with *A. thaliana* ACR2

**Figure 11:**
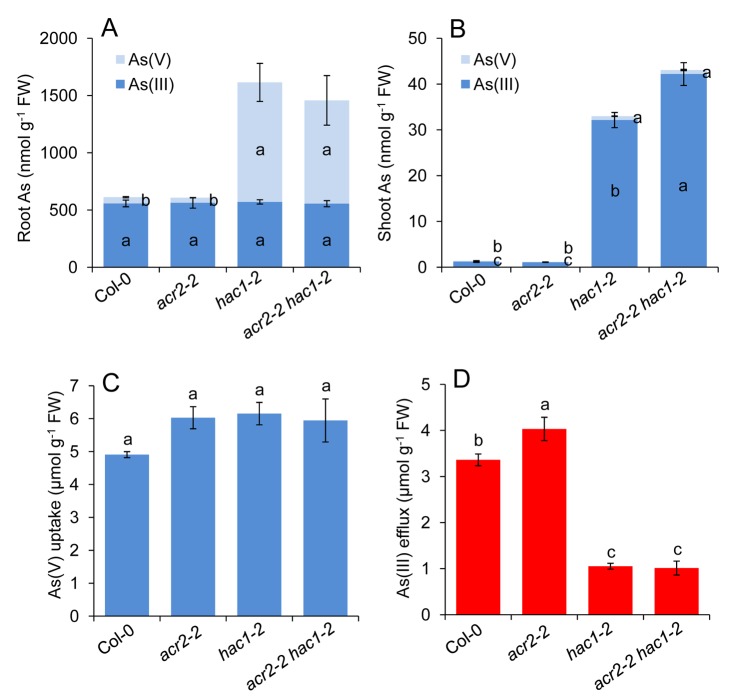
*HAC1* and *ACR2* do not interact additively as part of the metabolism of arsenic. Wild-type Col-0, single *acr2-2* and *hac1-2* mutants and the *acr2-2 hac1-2* double mutant were grown hydroponically for 3 wk and 5 µM arsenate were added for analysis thereafter. Accumulation of arsenate and arsenite was monitored in both roots (***A***) and shoots (***B***) for all genotypes. The uptake of arsenate (***C***) and efflux of arsenite (***D***) was also monitored and calculated from changes in their concentrations in the hydroponic growth media. Letters above bars indicate statistically different groups using a one-way ANOVA followed by least significant difference (LSD) test at the probability of *p*<0.05. Data represent means ± S.E. (*n* = 4). Raw data available in Data S9.

**Figure 12:**
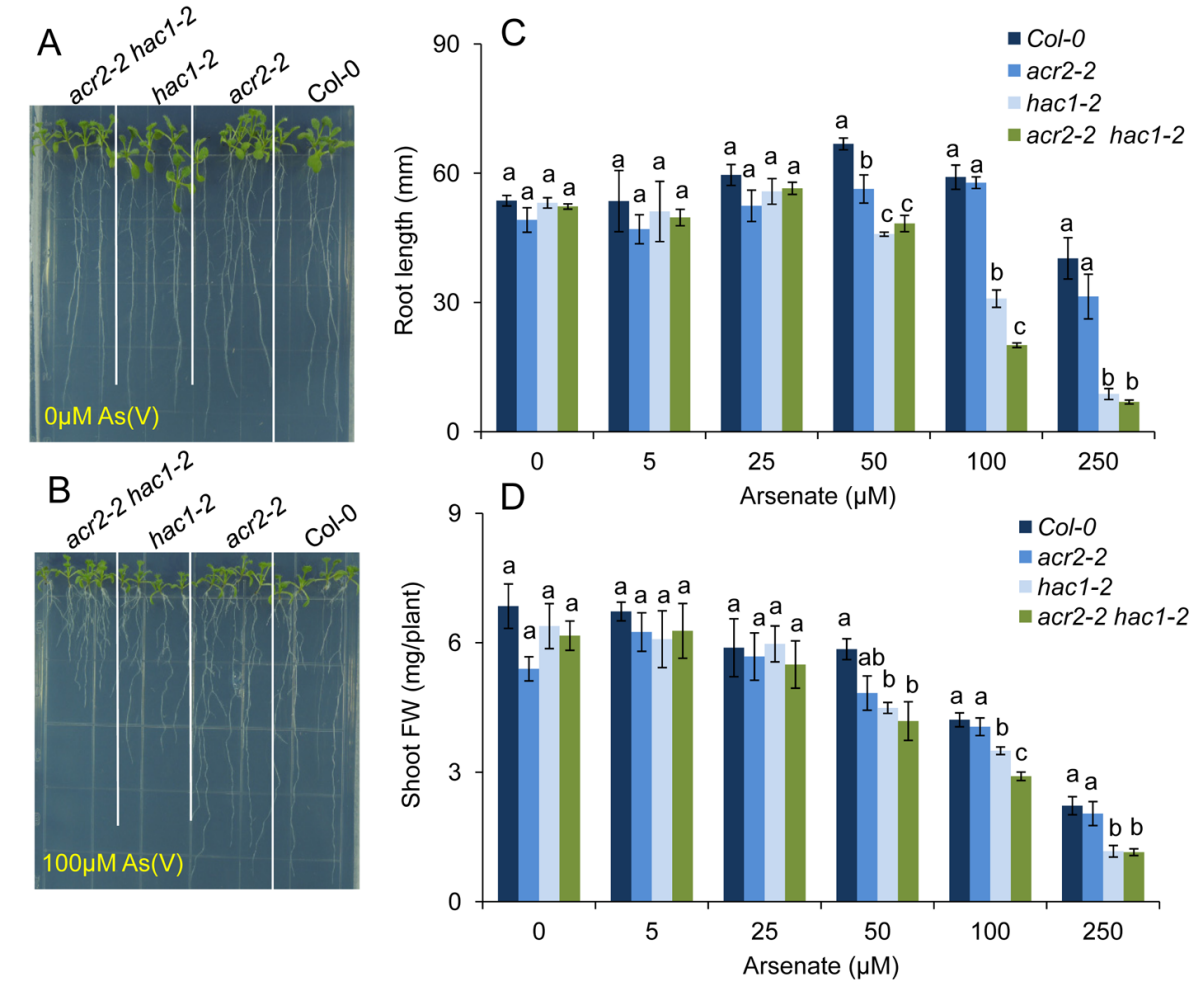
*HAC1* and *ACR2* do not interact epistatically as part of the arsenic resistance mechanism. Wild-type Col-0 and single *acr2-2* and *hac1-2* mutants and the *acr2-2 hac1-2* double mutant were grown on agar solidified nutrient medium containing 0 µM (***A***) and 100 µM (***C***) arsenate and after 12 days a representative photograph taken. Plant were also grown in the same conditions on nutrient medium containing a range of arsenate concentrations and the root length (***B***) and shoot fresh weight (***D***) determined after 12 days of growth. Letters above bars in (***B***, ***D***) indicate statistically different groups within treatments using a one-way ANOVA followed by least significant difference (LSD) test at the probability of *p*<0.05. Data represent means ± S.E. (*n* = 4). Raw data available in Data S10.

Previously, *ACR2* (also known as *CDC25*) from *A. thaliana* has been shown to encode an arsenate reductase when assayed in vitro [25]. We were, therefore, interested to know if *HAC1* and *ACR2* interact epistatically. To test this, we generated the double mutant *acr2 hac1* homozygous for loss-of-function alleles of both *ACR2* and *HAC1*. The *acr2-2 hac1-2* double mutant, along with both the parental single mutants and wild-type background, was exposed to arsenate in agar solidified growth medium or in hydroponic nutrient solution and the phenotypes associated with arsenic metabolism (arsenate uptake, arsenate reduction, arsenite efflux, arsenic accumulation, and arsenate resistance) evaluated. After exposure to 5 µM arsenate for 24 hr in the hydroponic medium, we observed in the *hac1-2* single mutant a significant increase in the accumulation of arsenate in roots (Figure 11A) and a significant decrease in arsenite efflux (Figure 11D), leading to a significant increase in total arsenic accumulation in shoots (Figure 11B) with no effect on arsenate uptake (Figure 11C). This is what we had previously observed for the single *hac1* mutants (Figure 3). Furthermore, loss-of-function of *ACR2* had no effect on the accumulation of arsenate in roots, efflux of arsenite or uptake of arsenate, or the total arsenic accumulation in shoots, as previously published [26]. Combining both *acr2-2* and *hac1-2* loss-of-function alleles in the *acr2-2 hac1-2* double mutant did not significantly alter either arsenate accumulation in roots or arsenite efflux compared to the *hac1-2* single mutant (Figure 11A, 11D). We did observe a significant increase in shoot arsenite accumulation in the *acr2-2 hac1-2* double (Figure 11B). However, this was not observed in the alternative *acr2-2 hac1-1* double mutant (Figure S5B), and we therefore conclude that *ACR2* and *HAC1* do not interact epistatically under the conditions in which we tested the phenotypes. Furthermore, at all arsenate concentrations tested no significant difference was observed in the growth (root length or overall fresh weight) of Col-0 wild type and *acr2-2* (Figure 12). This is similar to results previously published [26] and suggests that *ACR2* is not necessary for arsenate reduction or resistance under the conditions used in our experiments. As expected, loss-of-function of *HAC1* significantly reduced arsenate resistance compared to Col-0 wild type, measured as either root length or overall fresh weight (Figure 12) at concentrations of arsenate at and above 50 µM. However, combining both *acr2-2* and *hac1-2* loss-of-function alleles in the *acr2-2 hac1-2* double mutant did not significantly alter the arsenate resistance compared to the *hac1-2* single mutant (Figure 12). From these results, we conclude that under the conditions we tested *HAC1* and *ACR2* do not interact epistatically to affect arsenic metabolism or resistance. Furthermore, we reconfirm that *ACR2* plays no observable role in arsenic metabolism or resistance in vivo [26].

## Discussion

**Figure 13:**
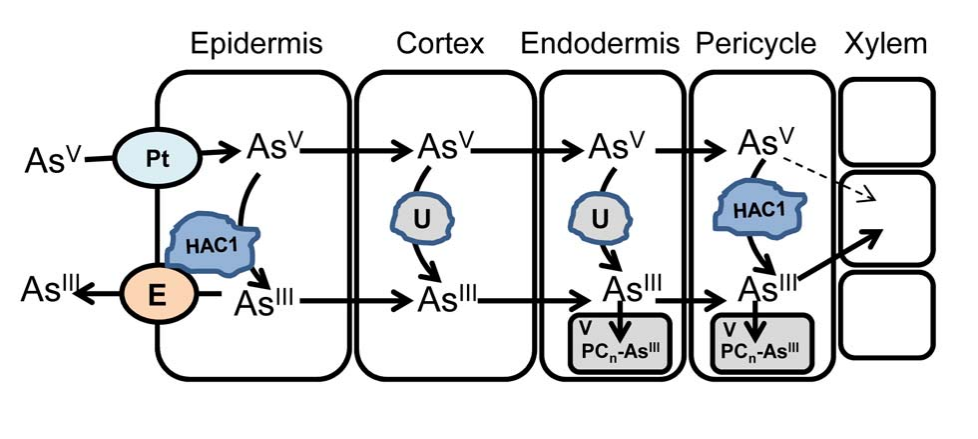
Schematic of the proposed role of HAC1 in arsenate metabolism in roots. A model and proposed function of HAC1 in the chemical transformations and transport processes arsenic undergoes during its radial transport from the soil, across the root and into the central vascular system for transport to the shoot. Pt, phosphate transporter; E, effluxer; U, unknown arsenate reductase; PC_n_-As^III^, phytochelatin-arsenite complex, V, vacuole.

Here, we have used genome-wide association mapping of loci associated with leaf arsenic accumulation to identify *HAC1*, a gene encoding a protein that plays a critical role in reducing arsenate to arsenite in roots to promote arsenite efflux as part of a plant's arsenic resistance mechanism (Figure 13). While this paper was in review, Sánchez-Bermejo and colleagues [64] also identified this gene, which they named *ATQ1*, as an arsenate reductase playing a critical role in arsenate resistance. However, in contrast to our study, these authors used QTL mapping of loci linked to variation in arsenate resistance in a biparental RIL population to identify *ATQ1*. We go beyond the findings of these authors by revealing the functional role of ATQ1 in arsenate resistance and investigating the role of HAC1/ATQ1 in arsenic accumulation. We find that natural variation at the *HAC1* locus accounts for a significant proportion of the species-wide diversity in leaf arsenic accumulation in *A. thaliana* when plants are grown in soil containing environmentally relevant trace concentrations of arsenic. Furthermore, we identify the *A. thaliana* accession Kr-0, collected from the Botanic Garden in Krefeld, Germany, as having a rare natural loss-of-function allele of *HAC1* that leads to extreme foliar accumulation of arsenic in this accession.

We show that the HAC1 protein accumulates in the root epidermis and root hairs, where it is ideally localised to play a critical role in the chemical reduction of arsenate to arsenite, a role necessary to allow arsenic, as arsenite, to be extruded from roots. Such efflux of arsenite is vital in order to control arsenic accumulation, which, left unchecked, can cause arsenic hyperaccumulation and toxicity. Arsenite efflux represents a large proportion of the arsenic taken up as arsenate by roots (Figure 8 and Figure 11) [26]. In the absence of functional HAC1 protein this efflux is abolished and arsenic over-accumulates in roots and shoots, leading to arsenic toxicity. In *E. coli* arsenate resistance is achieved in a similar manner to plants, by the reduction of arsenate to arsenite and efflux of arsenite from the cell [65]. We observed that the *HAC1* gene from *A. thaliana* is able to restore arsenate reduction capacity, arsenite efflux, and arsenate resistance to *E. coli* lacking their endogenous arsenate reductase (ArsC), in a similar manner to that recently shown by Sánchez-Bermejo and colleagues [64]. Furthermore, Sánchez-Bermejo and colleagues verified that the purified HAC1/ATQ1 recombinant protein has arsenate reductase activity [64]. These observations, taken together with the fact that arsenate accumulates in roots of plants lacking HAC1, strongly suggests that *HAC1* encodes an arsenate reductase enzyme.

In the absence of a functional HAC1 protein, *A. thaliana* still maintains the ability to reduce substantial amounts of arsenate to arsenite, suggesting that there are other arsenate reduction mechanisms in the plant. This redundancy has also been observed in *E. coli* [66] and *S. cerevisiae* [26]. One possibility is that arsenate could be reduced non-enzymatically by glutathione, though conversion rates might be slow. However, enzymes forming phosphorylated products can also promote arsenate reduction by incorrectly using arsenate in place of phosphate to generate arsenylated products in which the arsenate is more easily reduced by thiols such as glutathione [67]. Furthermore, both glutaredoxin and triosephosphate isomerise [68],[69] have also been suggested to promote arsenate reduction by as-yet-unknown mechanisms. More importantly, what is non-redundant for ArsC in *E. coli*, ACR2 in *S. cerevisiae*, and HAC1 in *A. thaliana* is their function as arsenate reductases enabling arsenite efflux and resistance to arsenate. Such observations suggest that these arsenate reductases are necessary for the reduction of arsenate to arsenite to create a specific pool of arsenite for efflux. It has been proposed [66] that this specificity may come about by the direct physical interaction of the arsenate reductase with the arsenite effluxer, thereby channelling arsenic for efflux. In support of this an actinobacterial enzyme containing an aquaglyceroporin-derived arsenite channel with a C-terminal arsenate reductase has been identified that provides single gene arsenate resistance [70]. The coupling of arsenate reduction and arsenite efflux could also occur by both proteins being localised to the same specific cellular location where arsenite produced by the reductase could be efficiently channelled to the efflux protein. In mutants lacking a functional *HAC1* non-specific arsenate reduction may occur at locations that do not contain the arsenite efflux protein, leading to arsenate reduction without efflux. Such suggested coupling of the HAC1, ArsC, and ACR2 arsenate reductases to their associated arsenite effluxer is quite different from the previously characterised *A. thaliana* arsenate reductase ACR2. This enzyme has been shown to have arsenate reductase activity as a purified enzyme in vitro [25]. However, loss-of-function of *ACR2* has no effect on arsenate reduction, arsenite efflux, arsenic accumulation or resistance to arsenate in vivo, as previous published [26] and repeated here. Furthermore, our genetic analysis using the *acr2 hac1* double mutant reveals that *ACR2* also does not interact epistatically with *HAC1*; supporting our conclusion that *ACR2* plays no role in arsenic metabolism in *A. thaliana*, even though in vitro ACR2 has arsenate reductase activity [25]. Our work goes beyond the recently published work of Sánchez-Bermejo and colleagues [64] in exploring experimentally the genetic interaction between *HAC1/ATQ1* and *ACR2*.

In the *hac1 A. thaliana* mutant that lacks the arsenate reductase activity required for arsenite efflux, we observe an over-accumulation of arsenic within the central stele of the root. This suggests that in the absence of sufficient arsenite efflux, due to a lack of appropriate arsenate reduction capacity, arsenate is transported radially across the root and accumulates within the stele (Figure 13). Once in the stele, this excess arsenate would be expected to load into the xylem and be translocated to the shoots, where it would be reduced to arsenite by *HAC1* independent mechanisms, leading to arsenic hyperaccumulation, as observed in the *A. thaliana hac1* mutant. Interestingly, we also observe the HAC1 protein to be localised to the pericycle close to the xylem within the stele. HAC1 in this location could provide the arsenate reduction capacity needed to maintain low concentrations of arsenate in the stele (Figure 13). Our work goes beyond the recently published work of Sánchez-Bermejo and colleagues [64] in developing such mechanistic insights. Since arsenate is known to act as a phosphate analogue, and can therefore be potentially loaded into the xylem via the phosphate transport system, limiting arsenate concentrations in the stele would be an effective mechanism to minimise arsenic translocation to the shoot. PHO1 is the main phosphate effluxer involved in xylem loading of phosphate [71],[72] in *A. thaliana*. Loss-of-function of *PHO1* does not reduce arsenic accumulation in shoots [73], and this supports the notion that in wild-type plants, with a functional HAC1 protein, arsenate concentrations in the stele are low enough that arsenate translocation does not contribute significantly to shoot arsenic accumulation [73]. In plants with a non-functional HAC1, the expected elevated translocation of excess arsenate does not appear to compete with phosphate transport, since leaf phosphate concentrations are equal in the wild type and *hac1* mutant (Figure S6). However, here plants were grown in soil containing only 0.1 µmoles g^−1^ dry weight arsenate and plants were fertilised regularly with nutrient solution containing 0.25 mM phosphate, making it unlikely that arsenate competition with phosphate for xylem loading could be detected. Competition may, however, be detectable if plants are exposed to high concentrations of arsenate, though such exposure would be toxic to the plant.

Loss-of-function of *HAC1* causes increased shoot accumulation of arsenic when plants are growing in soils containing only 7.5 mg/kg arsenic, a concentration well below many countries' clean-up guidelines for soil arsenic, which range from 0.039–40 mg/kg in the US (depending on the state) to 150 mg/kg in Japan (15 mg/kg limit applies to rice fields) [74]. This suggests that HAC1 functions constitutively to allow plants to maintain control over arsenic accumulation even when they are growing in soils that contain low arsenic concentration. However, *HAC1* expression is also induced by exposure to arsenate. This suggests that HAC1 is involved in both constitutive and plastic responses of *A. thaliana* to arsenate in the environment, allowing plants to maintain effective arsenite efflux from roots across a range of arsenic concentrations in the soil.

The discovery of *HAC1* now provides an explanation of why T-DNA insertion alleles of *ACR2* have been reported to not affect arsenic homeostasis in *A. thaliana* [26], whereas RNA interference has been shown to have a strong effect [23]. Since *ACR2* and *HAC1* share sequence identity (Figure S7) within the region used to knock down expression of *ACR2* by RNA interference [23], this sequence may also have suppressed *HAC1* expression. Such suppression of *HAC1* expression would then explain the enhanced arsenate sensitivity and arsenic hyperaccumulation observed by Dhankher and colleagues [23]; phenotypes that are the same as those we observed for the *hac1* mutants in *A. thaliana*.

Close to 18% of the total variation in leaf arsenic observed in the 349 *A. thaliana* accessions tested is explained by SNPs marking the linkage block that contains *HAC1*, and plants with the major allele of this locus contain, on average, 37% higher leaf arsenic concentration. The Kr-0 accession contains the major allele for this locus, and the elevated leaf arsenic in this accession is explained by the presence of a loss-of-function allele of *HAC1*. However, the nucleotide polymorphism in *HAC1^Kr-0^* that generates a non-functional HAC1 protein is not present in any other accession screened to date, establishing it as a rare allele. However, given that almost 75% of the accessions used in our GWA analysis contain the high leaf arsenic allele of the *HAC1* QTL, a significant amount of allelic diversity within the linkage block containing the *HAC1* gene remains to be characterised. The Kr-0 *HAC1* allele may therefore represent an extreme allele from within a larger set of *HAC1* alleles with less extreme variation in function. While this paper was in review, Sánchez-Bermejo and colleagues [64] reported on the existence of a second weak allele of *HAC1* (which they called *ATQ1*) from the Kas-1 accession. Unlike *HAC1* ^Kr-0^, which contains a premature stop codon, the Kas-1 allele contains multiple non-additive intra-allelic polymorphisms that lead to its reduced function. It is also interesting to note that since the majority of the *A. thaliana* accessions tested have the *HAC1* allele associated with higher leaf arsenic, they are likely to have weak alleles of *HAC1*. However, the selective benefit, if any, of having this weak allele of *HAC1* remains an open question.

The close coupling of arsenate reduction by HAC1 with arsenite efflux from roots suggests that both HAC1 and the arsenite effluxer [14] are closely associated through perhaps co-expression in the same cells and possibly via direct protein–protein interactions. Critically, it is this arsenite efflux process in roots that allows plants to maintain low arsenic concentrations in their shoots when they are exposed to environmentally relevant arsenate concentrations in the soil (Figure 13). The discovery of *HAC1* and its role in this process opens up new possibilities for the development of crop plants with reduced arsenic concentrations in their edible parts, potentially providing real benefits to human health by limiting arsenic intake in the diet. The existence of natural genetic variation at *HAC1* in *A. thaliana* holds out the promise that variation in HAC1 function may also exist in crops, providing the genetic material to develop low-arsenic-accumulating varieties. However, given the high frequency of the weak allele of *HAC1* across the *A. thaliana* species, it will be important to understand if there is any negative trade-off to having highly efficient arsenate reduction capacity.

## Methods

### Plant Material and Growth Conditions

The set of *A. thaliana* accessions used for this study contained 349 accessions selected from 5,810 worldwide accessions as previously described [44],[46]. T-DNA insertion mutants (GABI_868F11, SM_3_38332 for *hac1-1* and *hac1-2*, and GABI_Kat772G06 for *acr2-2*) were obtained from the Nottingham Arabidopsis Stock Centre (NASC). The plants used for analysis of leaf arsenic by ICP-MS were grown for 5 wk in a climate-controlled room with a photoperiod of 10 hr light (90 µmol m^−2^ s^−1^) 14 hr dark, humidity of 60% and temperature ranging from 19 to 22°C. The soil preparation, seed stratification, sowing, and plant cultivation followed protocols described previously [44],[46]. Plants used for analysis of the expression of *HAC1*, including quantitative real-time RT-PCR and for cell type and tissue expression pattern (*pHAC1::HAC1-GFP* transgenic lines), were grown in axenic conditions. In brief, seeds were surface sterilized with 50% bleach and 0.05%SDS for 15 min followed by being washed eight times with sterilized, deionized water and sown on 1/2 strength Murashige and Skoog (Sigma-Aldrich, St. Louis, US) media solidified with agar containing 1% sucrose in Petri dishes. Seeds on plates were stratified at 4°C for 3 days. Plates with seeds were then maintained at 16 hr light (90–120 µmol m^−2^ s^−1^) and 8 hr dark at 22°C. Hydroponic experiments were carried out as previously described [26]. Three-week-old plants were exposed to 5 µM arsenate for 24–48 hr. Arsenic species in the nutrient solution and in the roots and shoots were determined by HPLC-ICP-MS. The decrease of arsenate and the production of arsenite in the medium were used to calculate the arsenate uptake and arsenite efflux, respectively [26].

### Elemental Analysis

The concentration of total leaf arsenic was measured as ^75^As using inductively coupled plasma mass spectrometry (ICP-MS) as described previously [75]. Briefly, one to two adult rosette leaves were harvested from 5-week-old *A. thaliana* plants. The leaves were cleaned by rinsing with ultrapure water (18.2 MΩcm Milli-Q, Merck Millipore) and placed into Pyrex digestion tubes. Samples were dried in an oven at 88°C for 20 hours. After cooling, seven reference samples from each planted block were weighed. The samples, together with blank controls, were digested with 0.90 ml concentrated nitric acid (Baker Instra-Analyzed; Avantor Performance Materials) and diluted to 10.0 ml with ultrapure water (18.2 MΩcm). The internal standard Indium (In) was added to the acid prior to digestion for monitoring technical errors and plasma stability in the ICP-MS instrument. After samples and controls were prepared, elemental analysis was performed with an ICP-MS (Elan DRC II or NexION 300D; PerkinElmer) coupled to Apex desolvation system and SC-4 DX autosampler (Elemental Scientific Inc., Omaha, NE, US), monitoring these elements: Li, B, Na, Mg, P, S, K, Ca, Mn, Fe, Co, Ni, Cu, Zn, As, Se, Rb, Sr, Mo, and Cd. All samples were normalized to calculate weights, as determined with a heuristic algorithm using the best-measured elements, the weights of the seven weighed samples, and the solution concentrations [75], detailed at www.ionomicshub.org. For GWA analysis, data were normalised using common genotypes across experimental blocks as previously described [46],[76], and these normalised data have been deposited on the iHUB (previously known as PiiMS [77]) for viewing and download through www.ionomicshub.org.

### Association Mapping

Of the set of 349 *A. thaliana* accessions analyzed for leaf arsenic, a subset of 337 accessions were genotyped for 213,497 SNPs using the custom-designed SNP-tilling array Atsnptile 1 [44],[46],[78]. The GWA analysis was performed using a linear mixed model to correct confounding by population structure [79] implemented in the program EMMA (Efficient Mixed-Model Association) described previously [28].

### Positional Cloning

XAM was done as described previously [44],[59]. In brief, phenotyped F2 individuals were sorted by leaf arsenic concentration and approximately one quarter at each end of the F2 population distribution were pooled separately. Genomic DNA was extracted from the two pools and labelled separately using the BioPrime DNA labelling system (Invitrogen). The labelled DNA was hybridized to the Affymetrix SNP-tilling array Atsnptile 1. The CEL files containing raw data of signal intensity for all probes were read and spatially corrected using previously described R scripts [80] with the R program and the Bioconductor Affymetrix package. The original CEL files used in this study can be found in the Gene Expression Omnibus (GEO) under accession GSE62299. There are antisense and sense probes for each of the previously characterized polymorphic diallelic SNPs used here as genetic markers. The allele frequency difference between the two pools for each of these SNP markers was scored based on the signal intensity difference of the probes. The whole process was carried out using R scripts described previously [59].

The mapping interval by XAM was further narrowed down by PCR-based genotyping. Firstly, the 315 individuals of the F2 population used for XAM were genotyped individually at six cleaved-amplified polymorphic sequence (CAPS) markers as indicated in Figure 1C. Recombinants between marker CS85HKA and CS95HKB were selected for further analysis. The F2 recombinants with leaf arsenic concentration similar to Kr-0 were directly used for determination of the candidate region based on recombination between phenotype and genotypes at each marker, while the recombinants similar to Col-0 were selfed and the location of the candidate gene was determined based on recombination between phenotype and genotypes at each marker in the F3 progeny. After the rough mapping, three more polymorphic markers were developed within the rough mapping region between CS8901K and CS9249K, and an enlarged F2 population with 1,321 F2 individuals was used for fine mapping of the candidate gene. The candidate gene was further narrowed down using the same strategy as used for rough mapping. The primers and restriction enzymes for the CAPS markers are listed in Table S3.

### Sequencing of Candidate Genes

The *HAC1* genomic region of Kr-0 was sequenced using overlapping PCR as described previously [44]. Briefly, four pairs of primers for the PCR reactions were designed using Overlapping Primersets (http://pcrsuite.cse.ucsc.edu/Overlapping_Primers.html) (Table S3), and four overlapping fragments were amplified using these four pairs of primers with Kr-0 genomic DNA as the template. Each fragment was sequenced using its amplification primers in two directions. The sequenced reads were assembled using SeqMan Lasergene software (DNASTAR; http://www.dnastar.com), with the Col-0 sequence as the reference, and polymorphisms were identified by comparing the reference sequence and the Kr-0 sequence.

### Vector Construction, Transformation, Transgene, and Prokaryotic Expression

To construct the complementation vector of *HAC1*, a genomic DNA fragment including 1.49 kb promoter region, gene body and 0.34 kb 3′ downstream sequence was amplified by PCR from Col-0 using KOD hot start DNA polymerase (TOYOBO Bio-Technology, CO., LTD, Japan) and primer HAC-CU and HAC-CL (Table S3). The fragment was cloned into the pCR-XL-TOPO vector (Invitrogen Life Technologies) for sequencing and subsequently introduced into the binary vector pHB [81] using restriction enzymes *Eco*R I and *Pst* I to replace the 2×35S promoter. To construct the expression vector for expressing the fusion protein of HAC1-GFP driven by *HAC1* promoter, the *HAC1* genomic fragment including 1.49 kb promoter region and gene body with the stop codon replaced with TTA was PCR amplified from Col-0 using primer HAC-CU and HAC-GFP-Linker1 (Table S3). The *GFP* coding fragment was amplified from the pMDC vector using primer pair HAC-GFP-Linker2 and GFP-RP (Table S3). Thereafter, the fusion fragment of *pHAC1:HAC1-GFP* was amplified using the primer pair HAC-CU and GFP-RP with the above two PCR fragment products as template. The *pHAC1:HAC1-GFP* fragment was cloned into pCR-XL-TOPO vector for sequencing, and cloned into the pHB plant expression vector [81] using the *Eco*R I and *Pst* I restriction sites in pHB. The expression vectors were transformed into *Agrobacterium tumefaciens* strain GV3101 and introduced into Kr-0 and Col-0 using the floral dip method [82]. The transgenic lines were screened on half-strength Murashige and Skoog (Sigma-Aldrich, St. Louis, US) agar plates containing 50 µg/ml Hygromycin and 1% sucrose.

For prokaryotic expression of His-tagged *HAC1*, the full-length coding sequence of *HAC1* was amplified using the primers HAC-PEF and HAC-PER (Table S3) and cDNA products reverse-transcribed from Col-0 root RNA as the template. The fragment was cloned into T-easy vector (Promega). As the forward primer HAC-PEF introduced a *Nde* I restriction site, the insertion direction of the fragment in each clone was tested with the restriction enzyme *Nde* I. The clones with a *Nde* I site in the forward primer and a *Nde* I site in the multiple cloning site of the vector flanking the fragment were sequenced. The correct fragment was then cloned in-frame into the Nde I site of the prokaryotic expression vector pColD-TF (Takara, Japan), and verified by sequencing. The vector was transformed into *E. coli ΔarsC* mutant WC3110 (a strain lacking arsenate reductase activity) [62],[83] and its wild-type W3110 for complementation and assaying arsenate reductase activity. Expression of the His-tagged *HAC1* was induced with 1 mM IPTG at 16°C for at least 16 hr.

### Grafting of *A. thaliana* Plants

Reciprocal grafting was performed as previously described [44]. After graft unions established, grafted plants were examined under the stereoscopic microscope before transfer to potting mix soil to identify any adventitious root formation from the graft unions or above. Healthy grafted plants without adventitious roots were transferred to potting mix soil and grown in a controlled environment described above. After 4 wk, leaf samples were harvested for arsenic analysis. After harvesting, plants were examined again, and those with adventitious roots or without a clear graft union were removed from the subsequent analysis of the arsenic data.

### Arsenate Resistance Experiment and Arsenate Reductase Assay

The Δ*arsC* mutant WC3110 and its wild-type W3110 with *pColD-TF* empty vector or *pColD-TF-HAC1* were cultured at 37°C overnight. All cultured strains were diluted to OD600 nm = 0.5, and 50 µl inoculated into 5 ml of LB liquid media containing 1 mM IPTG and different concentrations of arsenate, as indicated in Figure 2. Cells were cultured at 16°C for 72 hr or at times indicated. The cell density was measured at OD600 nm using a spectrophotometer.

The *E. coli* bacteria strains for expressing His-tagged *HAC1* were lysed in arsenate reductase assay buffer (50 mM MES, 50 mM MOPS, pH 6.5, 300 mM NaCl, 0.1 mg/mL bovine serum albumin and 1% proteinase inhibitor cocktail [Sigma P9599]) using a French Press with a low temperature ultra-high pressure continuous flow cell disrupter (JNBIO Co., Ltd, China). The cell lysate was centrifuged at 16,000 g for 10 min at 4°C to remove cell debris and unbroken cells. Total protein concentration was measured using Coomassie Plus protein assay reagent (Pierce 23236). Arsenate reductase activity of the cell free extracts was measured using the previously established coupled assay [62]. Arsenite effluxed to the growth medium was measured by HPLC-ICP-MS.

### Arsenic Speciation Analysis

Plant samples were ground in liquid nitrogen to a fine powder in a mortar and pestle. The finely ground material (approximately 0.1 g) was extracted with 10 ml phosphate buffer solution (2 mM NaH_2_PO_4_ and 0.2 mM Na_2_-EDTA, pH 5.5) for 1 hr under sonication in a 4°C cold room [84]. The extract was filtered firstly through No. 42 Whatman filter paper and then through a 0.2 µm membrane filter. Arsenic speciation was determined using HPLC-ICP-MS (PerkinElmer NexION 300×, Waltham, MA, US). Arsenic species were separated using an anion-exchange column (Hamilton PRP X-100, fitted with a guard column; Reno, NV, US) with a mobile phase of 6.0 mM NH_4_H_2_PO_4_, 6.0 mM NH_4_NO_3_, and 0.2 mM Na_2_-EDTA (pH 6.2), run isocratically at 0.7 ml min^−1^. The solution from the separation column was mixed continuously with an internal standard solution (Indium) before being introduced into the ICP-MS. The instrument was set up in the kinetic energy discrimination mode with helium as the collision gas to reduce polyatomic interferences. Signals at m/z ^75^As and ^115^In were collected with a dwell time of 300 ms; the In counts were used to normalise the As counts. Arsenic species in the samples were quantified by external calibration curves using peak areas.

### Optical Microscopy

The *pHAC1::HAC1-GFP* transgenic *A. thaliana* lines grown in axenic condition for 1 wk were used for microscopic observation. GFP fluorescence of seedlings was observed using a stereo fluorescence microscope (M165FC, Leica) and a confocal laser microscope (FV-1000, OLYMPUS).

### Quantitative Real-Time RT-PCR

Total RNA extraction and cDNA synthesis were performed as described previously [44]. Quantitative real-time RT-PCR was done using a Real-Time PCR System ABI StepOnePlus (Life Technologies, US) using SYBR Green PCR Master Mix (Life Technologies, US) with the first strand cDNA as a template. Primers for quantitative RT-PCR (Table S3) were designed using Primer Express Software Version 3.0 (Life Technologies, US) with one primer of a pair covering an exon–exon junction. Expression data analysis was performed as previously described [44].

### Phylogenetic Analysis

Phylogenetic analyses were conducted using MEGA version6 [85]. Protein sequences were aligned using MAFFT7.1 [86] and the tree constructed using the parsimony method. Bootstraps were carried out with 1,000 replications. The GeneBank accession numbers for the protein sequences or the nucleotide sequences from which protein sequence were derived are: AY860059 (*OsACR2-1*), AY860058 (*OsACR2-2*), DQ310370 (*PvACR2*), BT003658.1 (*AtACR2*), BT008306.1 (*AtHAC1*), BAD07813.1 (*OsHAC1-1*), NP_001052310.1 (*OsHAC1-2*), NP_014928.1 (*ScHAC1*), CAB83305.1 (*AtACR2-2*), YP_005275964.1 (*AsrC*).

### Synchrotron μXRF

Three-week-old seedlings of Col-0, *hac1-1*, and *hac1-2* were exposed to 10 µM arsenate for 10 days in a hydroponic culture. Segments of roots at approximately 2 cm from the root tip were cut and placed into a planchette coated with hexadecane. The samples were frozen at −196°C with a pressure of 210 MPa for 30 s using a Leica HPM100 high pressure freezer [87]. The frozen samples were freeze substituted, embedded in resin, and sectioned into 7 µm thickness as previously described [87]. Synchrotron μXRF was undertaken at the Diamond Light Source on the I18 microfocus beamline. The incident X-ray energy was set to 12.4 keV using a Si(111) monochromator. The X-ray fluorescence spectra were collected using a Si drift detector. The beam size and step size were both 2 µm. Quantification of the concentrations of arsenic and other elements of interest in the samples were carried out using an external calibration with XRF reference materials.

## Supporting Information

Figure S1 ***HAC1*** **is not involved in limiting arsenic accumulation during exposure to arsenite.** When grown in hydroponic media containing 5 µM arsenite, both Kr-0 and Col-0 show no difference in arsenite or arsenate accumulation in shoots (***A***) and roots (***B***). No significant differences between geneotypes were observed using a one-way ANOVA followed by least significant difference (LSD) test at the probability of *p*<0.05. Data represent means ± S.E. (*n* = 4). Raw data available in Data S11.
(PDF)

Figure S2 **Multiple alignment of HAC1 orthologs in different plant species.** AlHAC, *Arabidopsis lyrata* HAC1, XP_002878530; AlHAC, *Arabidopsis lyrata* HAC1, XP_002878530; CrHAC1, *Capsella rubella* HAC1, XP_006296348; EsHAC1, *Eutrema salsugineum* HAC1, XP_006409311; BnHAC1, *Brassica napus* HAC1, CDY24335; EgHAC1, *Eucalyptus grandis*, KCW72671; PpHAC1, *Prunus persica*, XP_007217853; LjHAC1, *Lotus japonicas HAC1*, AFK36781; PtHAC1, *Populus trichocarpa* HAC1, XP_002321016; PmHAC1, *Prunus mume* HAC1, XP_008229745; MnHAC1, *Morus notabilis* HAC1, EXC35010; JcHAC1, *Jatropha curcas* HAC1, KDP29630; MdHAC1, *Malus domestica* HAC1, XP_008357978; FvHAC1, *Fragaria vesca subsp. Vesca* HAC1, XP_004303928; GmHAC1, *Glycine max HAC1*, NP_001241883; CaHAC1, Cicer arietinum HAC1, XP_004513263; StHAC1, *Solanum tuberosum* HAC1, XP_006347264; CsHAC1-1,*Cucumis sativus* HAC1-1, XP_004147071; CsHAC1-2, Citrus sinensis HAC1-2, XP_006486856; CmHAC1-1, *Cucumis melo* HAC1-1, XP_008457677; CmHAC1-2, *Cucumis melo* HAC1, XP_008457676; CmHAC1-3, *Cucumis melo* HAC1-3, XP_008442292; PvHAC1, *Phaseolus vulgaris HAC1*, XP_007131527; SlHAC1, *Solanum lycopersicum* HAC1, XP_004242121; CcHAC1, *Citrus clementina* HAC1, XP_006422627; MtHAC1-1, *Medicago truncatula* HAC1-1, AES65978; MtHAC1-2, *Medicago truncatula* HAC1-2, XP_003593790; MtHAC1-3, *Medicago truncatula* HAC1-3, KEH38050; MtHAC1-4, *Medicago truncatula* HAC1-4, XP_003595727.
(PDF)

Figure S3 **Fluorescence images of** ***HAC1*** **promoter driven HAC1-GFP fusion protein.** (***A***) Fluorescence image of whole transgenic seedling obtained with a stereo fluorescence microscope. (***B–C***) Fluorescence images of the root hair region (***B***) and root tip region (***C***) obtained with a confocal microscope. The upper panels in (***B*** and ***C***) show optical section at the position indicated with a green horizontal line, and the right panels in (***B*** and ***C***) show optical sections at the position indicated with a red vertical line. Blue lines in top and right panels represent the z-axis. Scale bar = 1 mm in (***A***) and 50 µm in (***B*** and ***C***).
(PDF)

Figure S4 ***HAC1*** **plays no role in arsenite resistance.** Both Col-0 wild type and the two *hac1* null alleles show no difference in shoot fresh weight (FW) after growth for 7 days in hydroponic Hoagland's solution containing various concentrations of arsenite. No significant differences between geneotypes were observed using a one-way ANOVA followed by least significant difference (LSD) test at the probability of *p*<0.05. Data represent means ± S.E. (*n* = 6). Raw data available in Data S12.
(PDF)

Figure S5 ***HAC1*** **and** ***ACR2*** **do not interact epistatically as part of the metabolism of arsenic.** Wild-type Col-0, single *acr2-2* and *hac1-1* mutants and the *acr2-2 hac1-1* double mutant were grown hydroponically for 3 wk and 5 µM arsenate added for analysis thereafter. Accumulation of arsenate and arsenite was monitored in both roots (***A***) and shoots (***B***) for all genotypes. The uptake of arsenate (***C***) and efflux of arsenite (***D***) was also monitored and calculated from changes in their concentrations in the hydroponic growth media. Letters above bars indicate statistically different groups using a one-way ANOVA followed by least significant difference (LSD) test at the probability of *p*<0.05. Data represent means ± S.E. (*n* = 4). Raw data available in Data S9.
(PDF)

Figure S6 **Loss-of-function of** ***HAC1*** **does not affect accumulation of phosphorus in shoots.** The concentration of total phosphorus was quantified by ICP-MS in leaves of wild-type Col-0, Kr-0, and *hac1-1* and *hac1-2* plants grown in artificial soil for 5 wk. Letters above bars indicate statistically different groups using a one-way ANOVA followed by least significant difference (LSD) test at the probability of *p*<0.05. Data represent means ± S.E. (*n* = 12). Raw data available in Data S13.
(PDF)

Figure S7 ***HAC1*** **and** ***ACR2*** **sequence similarity.** DNA Sequence similarity between *A. thaliana HAC1* and the 207 nucleotide sequence in the 3′ UTR of *ACR2* used for RNA interference in a previous study [23]. Numeric positions of nucleotides indicated above reference to the starting position of the *ACR2-RNAi* fragment [23], and below the sequences the numbers reference to the start codon for *A. thaliana HAC1*.
(PDF)

Table S1 **T-DNA insertion alleles of genes in the** ***HAC1*** **mapping interval.** To determine which gene is casual for *HAC1*, we analysed the arsenic concentration of leaves from 48 T-DNA insertion alleles of genes in the genetic mapping interval.
(XLSX)

Table S2 **Polymorphic sites between Kr-0 and Col-0 in At2g21045.** Two of these polymorphic sites are SNPs: one in an intron, and the other a synonymous SNP in an exon.
(XLSX)

Table S3 **Primers and restriction enzymes used in this study.**
(XLSX)

Data S1 **Raw data used to prepare** **Figure 1** **.**
(XLSX)

Data S2 **Raw data used to prepare** **Figure 2** **.**
(XLSX)

Data S3 **Raw data used to prepare** **Figure 3** **.**
(XLSX)

Data S4 **Raw data used to prepare** **Figure 4** **.**
(XLSX)

Data S5 **Raw data used to prepare** **Figure 6** **.**
(XLSX)

Data S6 **Raw data used to prepare** **Figure 7** **.**
(XLSX)

Data S7 **Raw data used to prepare** **Figure 8** **.**
(XLSX)

Data S8 **Raw data used to prepare** **Figure 10** **.**
(XLSX)

Data S9 **Raw data used to prepare** **Figure 11** **and Figure S5.**
(XLSX)

Data S10 **Raw data used to prepare** **Figure 12** **.**
(XLSX)

Data S11 **Raw data used to prepare Figure S1.**
(XLSX)

Data S12 **Raw data used to prepare Figure S4.**
(XLSX)

Data S13 **Raw data used to prepare Figure S6.**
(XLSX)

## Abbreviations

μ-XRF: synchrotron μX-ray fluorescence
ACR2: arsenic compounds resistance
Ann-1: Annecy-1
App1-16: Applerydvägen1-16
ArsC: arsenical resistanceC
*ATQ1*: *arsenate tolerance QTL*
B: boron
Benk-1: Bennekom-1
Bur-0: Burren-0
C: cytosine
CAPS: cleaved-amplified polymorphic sequence
Col-0: Columbia-0
DW: dry weight
EDTA: ethylenediaminetetraacetic acid
EMMA: efficient mixed-model association
GEO: gene expression omnibus
GFP: green fluorescent protein
GWA: genome-wide association
*HAC1*: *high arsenic content 1*
Hn-0: Hennetalsperre-0
HPLC-ICP-MS: high performance liquid chromatography–inductively coupled plasma–mass spectrometry
ICP-MS: inductively coupled plasma–mass spectrometry
iHub: ionomics hub
In: Indium
IPTG: isopropyl β-D-1-thiogalactopyranoside
Kas-1: Kashmir
Kr-0: Krefeld
MES: 2-(N-morpholino)ethanesulfonic acid
MOPS: 3-(N-morpholino)propanesulfonic acid
mRNA: messenger ribonucleic acid
NADPH: nicotinamide adenine dinucleotide phosphate
NASC: Nottingham Arabidopsis stock centre
*PHO1*: *phosphate1*
*PHT1;1*: *phosphate transporter1;1*
PiiMS: Purdue ionomics information management system
QTL: quantitative trait loci
RT-PCR: Reverse Transcription Polymerase Chain Reaction
RILs: recombinant inbred lines
RNAi: ribonucleic acid interference
SDS: sodium dodecyl sulfate
SNP: single nucleotide polymorphism
T: thymine
TAIR9: The Arabidopsis information resource 9 genome release
T-DNA: transfer deoxyribonucleic acid
WRKY6: WRKY transcription factor 6
XAM: extreme array mapping
