# Supplementary material for: Genome-wide Association Mapping Identifies a New Arsenate Reductase Enzyme Critical for Limiting Arsenic Accumulation in Plants
Source: PLoS Biol. 2014 Dec 2;12(12):e1002009. doi: 10.1371/journal.pbio.1002009 (PMC4251824; doi:10.1371/journal.pbio.1002009)
Supplement: Figure S1 — HAC1 is not involved in limiting arsenic accumulation during exposure to arsenite. When grown in hydroponic media containing 5 µM arsenite, both Kr-0 and Col-0 show no difference in arsenite or arsenate accumulation in shoots (A) and roots (B). No significant differences between geneotypes were observed using a one-way ANOVA followed by least significant difference (LSD) test at the probability of p<0.05. Data represent means ± S.E. (n = 4). Raw data available in Data S11. (PDF) [file pbio.1002009.s001.pdf]

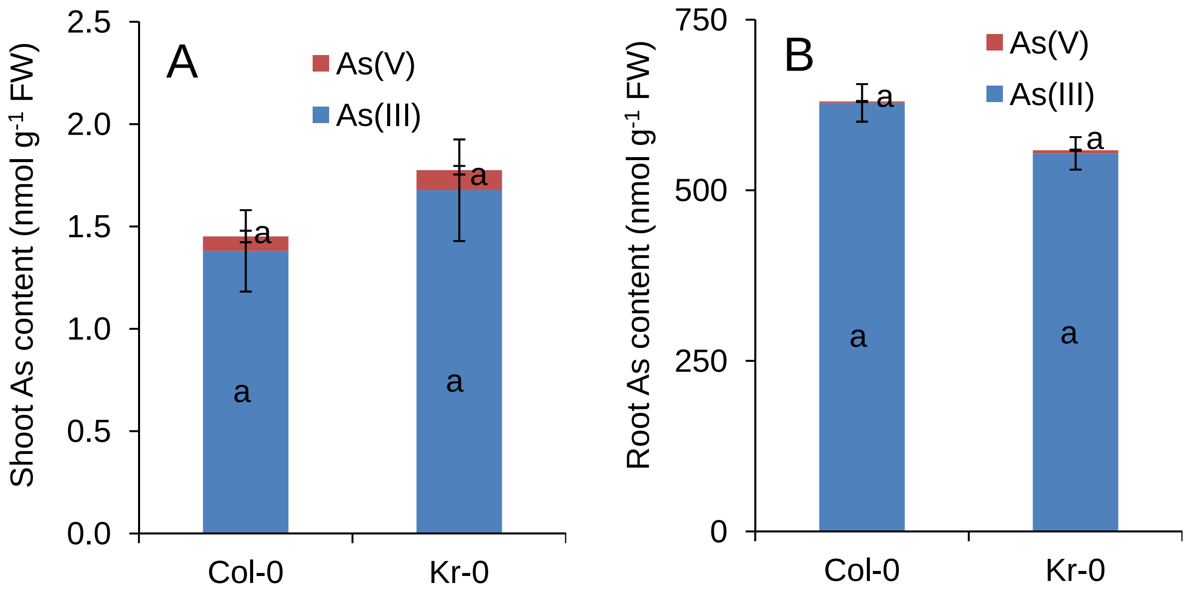

Figure S1. ***HAC1* is not involved in limiting arsenic accumulation during exposure to arsenite**. When grown in hydroponic media containing 5 µM arsenite wild-type Col-0 and Kr-0 with a loss-of-function allele of *HAC1* show no difference in arsenite or arsenate accumulation in shoots (**A**) and roots (**B**). No significant differences between genotypes were observed using a one way ANOVA followed by least significant difference (LSD) test at the probability of $p<0.05$. Data represent means ± S.E. (n = 4). Raw data available in Data S11.
