# Supplementary material for: Genome-wide Association Mapping Identifies a New Arsenate Reductase Enzyme Critical for Limiting Arsenic Accumulation in Plants
Source: PLoS Biol. 2014 Dec 2;12(12):e1002009. doi: 10.1371/journal.pbio.1002009 (PMC4251824; doi:10.1371/journal.pbio.1002009)
Supplement: Figure S2 — Multiple alignment of HAC1 orthologs in different plant species. AlHAC, Arabidopsis lyrata HAC1, XP_002878530; AlHAC, Arabidopsis lyrata HAC1, XP_002878530; CrHAC1, Capsella rubella HAC1, XP_006296348; EsHAC1, Eutrema salsugineum HAC1, XP_006409311; BnHAC1, Brassica napus HAC1, CDY24335; EgHAC1, Eucalyptus grandis, KCW72671; PpHAC1, Prunus persica, XP_007217853; LjHAC1, Lotus japonicas HAC1, AFK36781; PtHAC1, Populus trichocarpa HAC1, XP_002321016; PmHAC1, Prunus mume HAC1, XP_008229745; MnHAC1, Morus notabilis HAC1, EXC35010; JcHAC1, Jatropha curcas HAC1, KDP29630; MdHAC1, Malus domestica HAC1, XP_008357978; FvHAC1, Fragaria vesca subsp. Vesca HAC1, XP_004303928; GmHAC1, Glycine max HAC1, NP_001241883; CaHAC1, Cicer arietinum HAC1, XP_004513263; StHAC1, Solanum tuberosum HAC1, XP_006347264; CsHAC1-1,Cucumis sativus HAC1-1, XP_004147071; CsHAC1-2, Citrus sinensis HAC1-2, XP_006486856; CmHAC1-1, Cucumis melo HAC1-1, XP_008457677; CmHAC1-2, Cucumis melo HAC1, XP_008457676; CmHAC1-3, Cucumis melo HAC1-3, XP_008442292; PvHAC1, Phaseolus vulgaris HAC1, XP_007131527; SlHAC1, Solanum lycopersicum HAC1, XP_004242121; CcHAC1, Citrus clementina HAC1, XP_006422627; MtHAC1-1, Medicago truncatula HAC1-1, AES65978; MtHAC1-2, Medicago truncatula HAC1-2, XP_003593790; MtHAC1-3, Medicago truncatula HAC1-3, KEH38050; MtHAC1-4, Medicago truncatula HAC1-4, XP_003595727. (PDF) [file pbio.1002009.s002.pdf]

```
                     *        20         *        40         *        60         *        80
AtHAC1   : MYTYSLLNLSHCRRQTRKKRKTDHTEGFLMEETKPKTVEDVETVDVYTAKGFL-S--TGHRYLDVRTNEEFAKSHVEEAL : 77
OsHAC1-1 : ----------------------MAPPYETSAAGSESP-VPVVTVDVAAASDLITS--AGHRYVDVRTEEEMNKGHLHNSL : 55
OsHAC1-2 : -------MMMMVRLPAMFVICILAVPLLP-ALGSEPPSTPVPTVGVTAASHLVGS--GGHSYLDVRTEEEFKKGHVENSL : 70
AlHAC1   : -----------------------------MEETKPKTVEDVETVDVYTAKGFL-S--TGHRYLDVRTNEEFAKSHVEDAL : 48
CrHAC1   : -----------------------------MEETKTKTVENVETVDVYTAKGLIST--TGHRYLDVRTNEEFAKSHFEEAL : 49
EsHAC1   : -----------------------------METPKTKTVEDVENVDVYTAKGLI-T--VGHRYVDVRTNEEFAKSHFEDAL : 48
BnHAC1   : -----------------------------MEKTNTKTFEDVESVDVYTAKGLI-TI-GHHRYLDVRTNEEFAKSHFDDAL : 49
EgHAC1   : -----------------------------METIKKS--EDVANVDVYTAKGLI-T--VGHRYLDVRTEEEFKKSHVENAV : 46
PpHAC1   : -----------------------------MAATKRP--GDVASVDVYTAKGLI-S--VGHRYLDVRTVEEFNKSHVENAL : 46
FmHAC1   : -----------------------------MAATKRP--EDVASVDVYTAKGLI-S--VDHRYLDVRTVEEFNKSHVENAL : 46
MdHAC1   : -----------------------------MAAMKGP--EDVASVDVYTAKGLI-S--IGHRYLDVRTIEEFNKSHVENAM : 46
FvHAC1   : -----------------------------MAEAKRG--EDVKSVDVFTAKGLI-S--TGHLYLDVRTSEEFNTSHIENAI : 46
MnHAC1   : -----------MKVNACRVKCQYFNKSTNMHSTTSA--EDVVNVDVYSVKGLI-S--VGHRYLDVRTPEEFNKSHVDGAF : 64
PtHAC1   : -----------------------------MDATQRP--QDVITVDVHAAKGLI-A--SGHRYLDVRTAEEFNKSHVDNAL : 46
JcHAC1   : -----------------------------MDATKRP--EDVVTVDVQAAKDLI-S--SGYYYLDVRTTEEFNKSHIENAL : 46
CcHAC1   : -----------------------------MDAIKKP--EDVASVDVHAAKDLIG---SGHRYLDVRTTAEFKKGHVDKAL : 46
MtHAC1-3 : -----------------------------MDMKKDH--QDVVTLDVHATKDLIDS--SGYNYLDVRTVEEFNKSHVENAI : 47
MtHAC1-4 : -----------------------------MDMKKDH--QDVVTLDVHATKDLIDS--SGYNYLDVRTVEEFNKSHVENAI : 47
MtHAC1-1 : -----------------------------MDMKKDH--QDVVTLDVHATKDLIDS--SGYNYLDVRTVEEFNKSHVENAI : 47
MtHAC1-2 : -----------------------------MDTTKDH--QNVVTLDVHAAKDLIHS--SGYNYLDVRSVEEFNKSHVENAI : 47
CaHAC1   : -----------------------------MDATKDH--QNILTLDVHAAKDLIDS--SGYHYLDVRSVEEFKKSHVENAI : 47
GmHAC1   : -----------------------------MDSPKDH--QNVVTINVRAAKDLINS--SGYRYLDVRSVEEFNKSHVENAH : 47
PvHAC1   : -----------------------------MDATKDH--QNVVTIDVHAAKNLINS--SDYSYLDVRSVEEFNKSHVENAY : 47
LjHAC1   : -----------------------------MDAPKDH--QDVVNVDVEAAKDLINS--SGYRYLDVRTVEEFNKSHVDNAL : 47
StHAC1   : -----------------------------MDSIKSN--EDVSNVDVISAKDLI-S--LGHTYLDVRTVEEYNRGHIDKAI : 46
SlHAC1   : -----------------------------MDSIKSN--EDVSNVDVISAKDLI-S--SGHTYLDVRTTEEYNRGHIDKAI : 46
CmHAC1-1 : -----------------------------MER----------TVDVQVAKDLIE---KGHLYLDVRTVEEYNKGHVENAL : 38
CmHAC1-2 : -----------------------------MER----------TVDVQVAKDLIE---KGHLYLDVRTVEEYNKGHVENAL : 38
CsHAC1-1 : -----------------------------MER----------TVDVQVAKDLIE---KGRLCLDVRTVEEYNKGHVENAL : 38
CmHAC1-3 : -----------------------------MER----------MVDVQVAKDLIE---KGHLYLNVKTVEEDNKGHVENAL : 38
CsHAC1-3 : -----MMSCRNIFALTKLRKNSFIPIRTIIDVGKRP-AGDVASVGVDTAKDLI-S--SGHRFLDVRTTEEFNESHVHGAL : 71
ScHAC1   : -------------------MWKAVMNAWNGTESQSKNVSNIQSYSFEDMKRIVGKHDPNVVLVDVREPSEYSIVHIPASI : 61
                                                        v  ak  6     g   61V4  eE    H   a
```

continue

```
                    *       100         *       120         *       140         *       160
AtHAC1   : NIPYMFKTDEG--------RVINPDFLSQVASVCK-KDEHLIVACNAGGRGSRACVDLLNEG----------------YD : 132
OsHAC1-1 : NVPFMFVTPQG--------REKNPLFVEQFSSLVS-KEEHVVVGCQSGKRSELACVDLLEAG----------------FK : 110
OsHAC1-2 : NVPFLFFTPQG--------KEKNTKFIEQVALHYD-KEDNIIVGCLSGVRSELASADLIAAG----------------FK : 125
AlHAC1   : NIPYMFQTDEG--------RVINPDFLPQVASVCK-KDEHMIVACNAGGRGSRACVDLLNAG----------------YE : 103
CrHAC1   : NIPYMFKTDEG--------RVINPDFLPQVASVCK-KDEHLIVACNSGGRASRACVDLLNAG----------------YE : 104
EsHAC1   : NIPYMFKTDEG--------RVVNPDFLSQVASGCK-KDDHLIVACNSGGRASRACVDLLNAG----------------YE : 103
BnHAC1   : NIPYMFKTDEG--------RIVNPDFLPQVASVCK-KDDNLIVACNSGGRATRACVDLLNAG----------------YE : 104
EgHAC1   : NVPYMFSTPDG--------RVKNPDFLTQVAAVCN-KEDHLVVGCNSGGRSLRACVDLLNAG----------------YK : 101
PpHAC1   : NIPYMFITEEG--------RVKNPEFLTQISSILK-KQDHLVVGCNSGGRSLKACVDLLNEG----------------FQ : 101
FmHAC1   : NIPYMFITEEG--------RVKNHEFLTKISSILK-KQDHLVVGCNSGGRSLKACVDILNEG----------------FQ : 101
MdHAC1   : NIPFMLITEEG--------RVKNPEFLTQISSTLK-KEDHLVVGCNSGGRALKACVDLLNEG----------------FQ : 101
FvHAC1   : NVPYMFKTQEG--------KVKNPEFLIRISSILK-KQDHLVVGCNSGGRSLKACVDLLNEG----------------FE : 101
MnHAC1   : NVPYMFITQEG--------RVENPDFLSQVSAICK-KDDHLVVGCNSGGRARRACVNLLNAG----------------FE : 119
PtHAC1   : NVPFMFKTDEG--------RVKNPEFLSKVASICS-KDDYLVVGCNSGGRSLRACIDLLGAG----------------FE : 101
JcHAC1   : NVPYMFKTQQG--------RVNNPQFLSLVASICK-KHDYLVLGCNSGGRSLRACVDLLNAG----------------FE : 101
CcHAC1   : NAPYI-KSE------------KNPEFLTQVASLCK-KEDPSIVVCNSGGRALRACVDLRNA-------------------  :  93
MtHAC1-3 : NVPYLFSTEEG--------RVKNPDFVNQVEAIYK-SEDHLIVACNAGGRSSRAWVDLHNS-------------------  :  99
MtHAC1-4 : NVPYLFSTEEG--------RVKNPDFVNQVEAIYK-SEDHLIVACNAGGRSSRAWVDLHNSERIGDDNKWILTLLLLGFK : 118
MtHAC1-1 : NVPYLFSTEEG--------RVKNPDFVNQVEAIYK-SEDHLIVACNAGGRSSRAWVDLHNS----------------GFK : 102
MtHAC1-2 : NVPYMFKTEEG--------RVKNPDFVNQVAAICK-SEDHLIVACNSGGRSIRACVDLHNS----------------GFQ : 102
CaHAC1   : NVPYMFITEAG--------RVQNPDFVKQVAEIYK-SEDHLIVACNSGGRSSRACVDLLNS----------------GYE : 102
GmHAC1   : NVPYVFITEAG--------RVKNPDFVDQVAAICK-TEDHLIVACNSGGRSLRASVDLLDS----------------GFK : 102
PvHAC1   : NVPYMFITEAG--------RVKNPDFVEQVTAICK-NEDHLIVACNSGGRSLKASVDLLDS----------------GFK : 102
LjHAC1   : NTAYMFSTEAG--------RVKNPDFLDQVAAVCK-SEDHLLVACNSGGRGRKAVIDLLDS----------------GYK : 102
StHAC1   : NIPYMFLNEQG--------RVKNPDFLEQVCSVCQ-KEDHLIVGCNSGGRGLRACVDLLNAG----------------YK : 101
SlHAC1   : NIPYMFLNEQG--------RVKNPDFLEQVSAVCQ-KEDHLIVGCNSGGRGLRACVDLLNAK----------------VY : 101
CmHAC1-1 : NVPYMFLTPEG--------RVKNPDFLAQVTSILK-KEDHIVVACNSGGRGLRACVDLLNAG----------------FE :  93
CmHAC1-2 : NVPYMFLTPEGSNKWSSTCRVKNPDFLAQVTSILK-KEDHIVVACNSGGRGLRACVDLLNAG----------------FE : 101
CsHAC1-1 : NVPYVFFTPEG--------QVKNPDFLAQVTSILK-KEDHIVVNCNRGGRGLRACVDLLNAG----------------FE :  93
CmHAC1-3 : NVSYMFLTPEG--------RVKNPDFLAQATSILK-KEDHIVAACNSGGRGLHACVDLLNA-------------------  :  90
CsHAC1-3 : NVPYLFITQEG--------RVKNPEFLTQVASVCS-KEDHIIVGCKSGIRSLCACVDLLNAG----------------YE : 126
ScHAC1   : NVPYRSHPDAF--------ALDPLEFEKQIGIPKPDSAKELIFYCASGKRGGEAQKVASSHG----------------YS : 117
           N p5 f    g           np F  q         dh 6  CnsGgR   A
```

continue

```
                     *         180         *         200
AtHAC1    : HVANMGGGYSAWVDAGFAG-DK--PPEDLKIACKFRPKEN------- : 169
OsHAC1-1  : NVKNMGGGYAAWLDNGFPINT---PPHTMY----------------- : 137
OsHAC1-2  : NVKNMEGGYMAWVENGLAVNK---PLVQEE----------------- : 152
AlHAC1    : HVANMGGGYSAWVDAGFAG-DK--PREELKIACQFRPKEN------- : 140
CrHAC1    : HVANMGGGYSAWVDAGFAGE-K-TP-EDLKIACKFRPNDN------- : 141
EsHAC1    : HVANMEGGYSAWVDAGFAG-DK--PPGELKIACKFRPKDN------- : 140
BnHAC1    : HVANMEGGYSAWVDAGFAG-DK--PAAELKTACKFRPKDN------- : 141
EgHAC1    : NVNNMEGGYSAWVDKGFAG-DK--PAEELKTACKFRP---------- : 135
PpHAC1    : HVTNMEGGYSAWVDSELAH-DK--PTEDLKVACKFRP---------- : 135
FmHAC1    : HVTNMEGGYSAWVDSGLAH-DK--PTEDLKVACKFRP---------- : 135
MdHAC1    : HVTNXEGGYSSWVDSGLAG-DK--PSDELKVACKFRP---------- : 135
FvHAC1    : HVTNMERGYSSWVDNGHAG-DKDKPSDELKIACKFRP---------- : 137
MnHAC1    : HVNSMEGGYSKWVDSGLAG-DK--PAETLKTACKFRP---------- : 153
PtHAC1    : HVTNMEGGYSAWVDSGFAG-DK--PAEELKTFCKFRP---------- : 135
JcHAC1    : HVRNMEGGYSAWVDSGFAGEDK-QP-EQLKTSCKFRP---------- : 136
CcHAC1    : HVTKLEGGYSAWVDEGVPG-DK--PLEELKISCKFR----------- : 126
MtHAC1-3  : -VSL------------------------------------------- : 102
MtHAC1-4  : HIVNMGGGYSAWVDAGFAGDDK--PAEELKTSCKFRP---------- : 153
MtHAC1-1  : HIVNMGGGYSAWVDAGFAGDDK--PAEELKTSCKFRP---------- : 137
MtHAC1-2  : HIVNMGGGYSAWVDAGFAGDDK--PANELKTACKFRP---------- : 137
CaHAC1    : HIINMGGGYSGWVDAGFAG-NI--PAEELKTSCKFRP---------- : 136
GmHAC1    : NIVNMGGGYSAWVDAGFAG-NK--PGEDLKTSCKFRPNIAKPTAQP : 145
PvHAC1    : HIVNMGGGYSAWVDAGFAG-DK--PPEELKTSCKIRR---------- : 136
LjHAC1    : HVFNMGGGYSAWVDAGFAG-DK--PAEELKTACKFRP---------- : 136
StHAC1    : DVRNLEGGYSAWVDNEFKGDEA---AQQFKTACKFRP---------- : 135
SlHAC1    : SNANISG---------------------------------------- : 108
CmHAC1-1  : HVNNMGGGYSAWVDSGFAGE-K--PPEELKIACKFRG---------- : 127
CmHAC1-2  : HVNNMGGGYSAWVDSGFAGE-K--PPEELKIACKFRG---------- : 135
CsHAC1-1  : HVNNMGGGYSAWVDSGFAGE-K--PPEELKIACKFRGSLS------- : 130
CmHAC1-3  : -VR----------------------FEMLELICKVD----------- : 103
CsHAC1-3  : HVTSMEGGYSAWLDKELAEDNK--PAAELKTC--------------- : 156
ScHAC1    : NTSLYPGSMNDWVSHG--G-DK------IDL---------------- : 139
               n  ggys w d g        p    k  c
```

continue

Figure S2. Multiple alignment of HAC1 orthologs in different plant species. AlHAC, *Arabidopsis lyrata* HAC1, XP_002878530;AlHAC, *Arabidopsis lyrata* HAC1, XP_002878530; CrHAC1,*Capsella rubella* HAC1, XP_006296348; EsHAC1, *Eutrema salsugineum* HAC1, XP_006409311; BnHAC1,*Brassica napus* HAC1, CDY24335; EgHAC1,*Eucalyptus grandis*, KCW72671; PpHAC1,*Prunus persica*, XP_007217853; LjHAC1,*Lotus japonicas HAC1*, AFK36781; PtHAC1,*Populus trichocarpa* HAC1, XP_002321016; PmHAC1,*Prunus mume* HAC1, XP_008229745; MnHAC1,*Morus notabilis* HAC1, EXC35010; JcHAC1,*Jatropha curcas* HAC1, KDP29630; MdHAC1,*Malus domestica* HAC1, XP_008357978; FvHAC1,*Fragaria vesca subsp. Vesca* HAC1, XP_004303928; GmHAC1,*Glycine max HAC1*, NP_001241883; CaHAC1,Cicer arietinum HAC1, XP_004513263; StHAC1,*Solanum tuberosum* HAC1, XP_006347264; CsHAC1-1,*Cucumis sativus* HAC1-1, XP_004147071; CsHAC1-2,Citrus sinensis HAC1-2, XP_006486856; CmHAC1-1,*Cucumis melo* HAC1-1, XP_008457677; CmHAC1-2,*Cucumis melo* HAC1, XP_008457676; CmHAC1-3,*Cucumis melo* HAC1-3, XP_008442292; PvHAC1,*Phaseolus vulgaris HAC1*, XP_007131527; SlHAC1,*Solanum lycopersicum* HAC1, XP_004242121; CcHAC1,*Citrus clementina* HAC1, XP_006422627; MtHAC1-1,*Medicago truncatula* HAC1-1, AES65978; MtHAC1-2, *Medicago truncatula* HAC1-2, XP_003593790;MtHAC1-3,*Medicago truncatula* HAC1-3, KEH38050; MtHAC1-4,*Medicago truncatula* HAC1-4, XP_003595727.
