# Supplementary material for: Genome-wide Association Mapping Identifies a New Arsenate Reductase Enzyme Critical for Limiting Arsenic Accumulation in Plants
Source: PLoS Biol. 2014 Dec 2;12(12):e1002009. doi: 10.1371/journal.pbio.1002009 (PMC4251824; doi:10.1371/journal.pbio.1002009)
Supplement: Figure S3 — Fluorescence images of HAC1 promoter driven HAC1-GFP fusion protein. (A) Fluorescence image of whole transgenic seedling obtained with a stereo fluorescence microscope. (B–C) Fluorescence images of the root hair region (B) and root tip region (C) obtained with a confocal microscope. The upper panels in (B and C) show optical section at the position indicated with a green horizontal line, and the right panels in (B and C) show optical sections at the position indicated with a red vertical line. Blue lines in top and right panels represent the z-axis. Scale bar = 1 mm in (A) and 50 µm in (B and C). (PDF) [file pbio.1002009.s003.pdf]

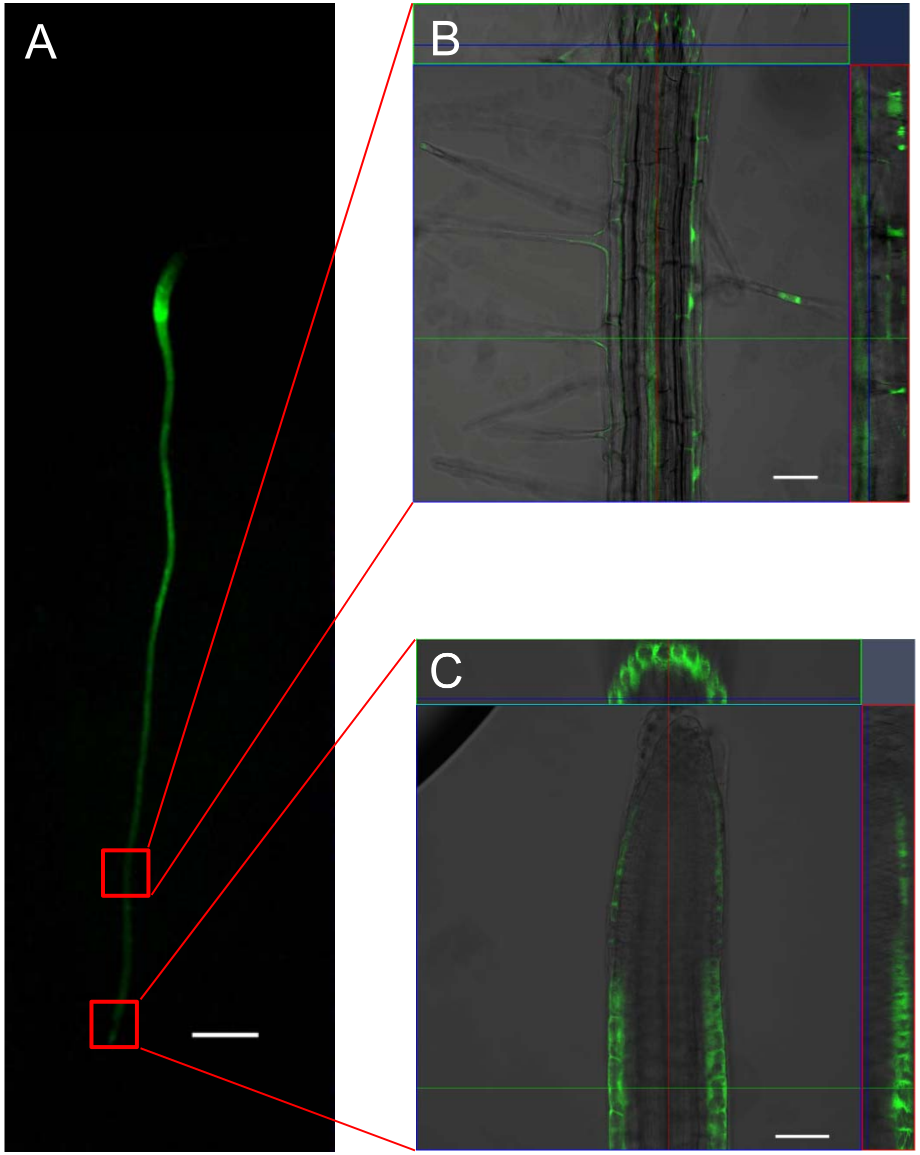

Figure S3. Fluorescence images of *HAC1* promoter driven HAC1-GFP fusion protein. (***A***) Fluorescence image of whole transgenic seedling obtained with a stero fluorescence microscope. (***B-C***) Fluorescence images of the root hair region (***B***) and root tip region (***C***) obtained with a confocal microscope. The upper panels in (***B-C***) show optical section at the position indicated with a green horizontal line, and the right panels in (***B-C***) show optical sections at the position indicated with a red vertical line. Blue lines in top and right panels represent the z-axis. Scale bar = 1 mm in (**A**) and 50 μm in (**B** and **C**).
