# Supplementary material for: Genome-wide Association Mapping Identifies a New Arsenate Reductase Enzyme Critical for Limiting Arsenic Accumulation in Plants
Source: PLoS Biol. 2014 Dec 2;12(12):e1002009. doi: 10.1371/journal.pbio.1002009 (PMC4251824; doi:10.1371/journal.pbio.1002009)
Supplement: Figure S4 — HAC1 plays no role in arsenite resistance. Both Col-0 wild type and the two hac1 null alleles show no difference in shoot fresh weight (FW) after growth for 7 days in hydroponic Hoagland's solution containing various concentrations of arsenite. No significant differences between geneotypes were observed using a one-way ANOVA followed by least significant difference (LSD) test at the probability of p<0.05. Data represent means ± S.E. (n = 6). Raw data available in Data S12. (PDF) [file pbio.1002009.s004.pdf]

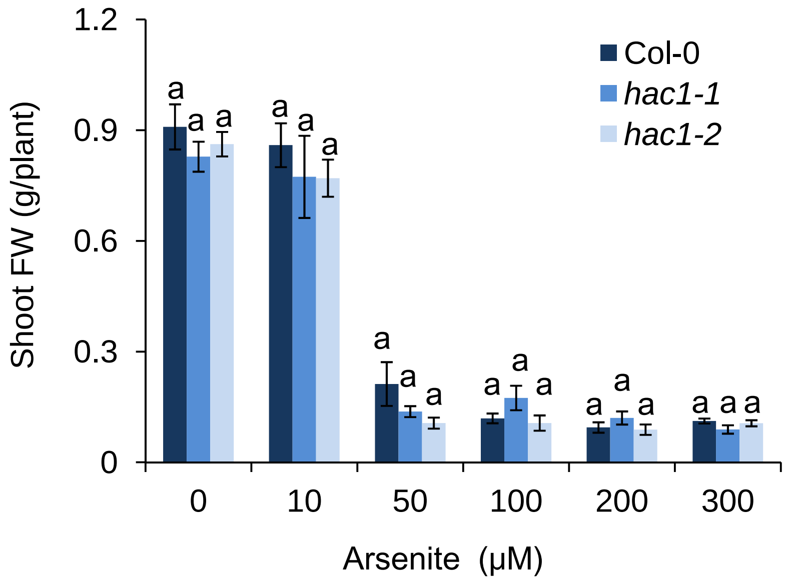

Figure S4. ***HAC1* plays no role in arsenite resistance**. Both Col-0 wild-type and the two *hac1* null alleles show no difference in shoot fresh weight (FW) after growth for 7 days in hydroponic Hoagland's solution containing various concentrations of arsenite. No significant differences between geneotypes were observed using a one way ANOVA followed by least significant difference (LSD) test at the probability of $p<0.05$. Data represent means ± S.E. (n = 6). Raw data available in Data S12.
