# Supplementary material for: Genome-wide Association Mapping Identifies a New Arsenate Reductase Enzyme Critical for Limiting Arsenic Accumulation in Plants
Source: PLoS Biol. 2014 Dec 2;12(12):e1002009. doi: 10.1371/journal.pbio.1002009 (PMC4251824; doi:10.1371/journal.pbio.1002009)
Supplement: Figure S5 — HAC1 and ACR2 do not interact epistatically as part of the metabolism of arsenic. Wild-type Col-0, single acr2-2 and hac1-1 mutants and the acr2-2 hac1-1 double mutant were grown hydroponically for 3 wk and 5 µM arsenate added for analysis thereafter. Accumulation of arsenate and arsenite was monitored in both roots (A) and shoots (B) for all genotypes. The uptake of arsenate (C) and efflux of arsenite (D) was also monitored and calculated from changes in their concentrations in the hydroponic growth media. Letters above bars indicate statistically different groups using a one-way ANOVA followed by least significant difference (LSD) test at the probability of p<0.05. Data represent means ± S.E. (n = 4). Raw data available in Data S9. (PDF) [file pbio.1002009.s005.pdf]

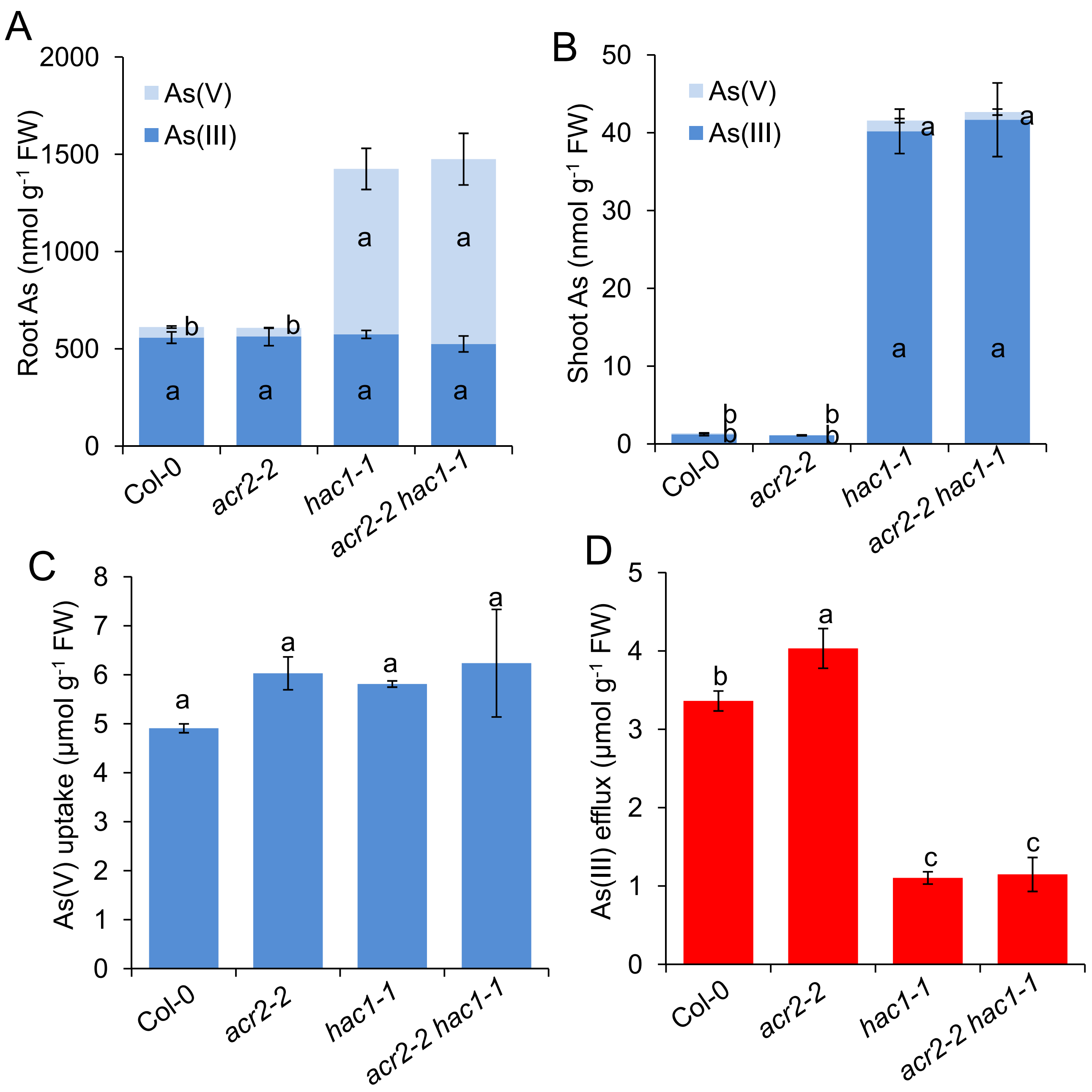
A
2000
1500
1000
500
0
Root As (nmol g-1 FW)
As(V)
As(III)
b
b
a
a
a
a
a
a
Col-0
acr2-2
hac1-1
acr2-2 hac1-1
B
50
40
30
20
10
0
Shoot As (nmol g-1 FW)
As(V)
As(III)
b
b
b
b
a
a
a
a
Col-0
acr2-2
hac1-1
acr2-2 hac1-1
C
8
7
6
5
4
3
2
1
0
As(V) uptake (μmol g-1 FW)
a
a
a
a
Col-0
acr2-2
hac1-1
acr2-2 hac1-1
D
5
4
3
2
1
0
As(III) efflux (μmol g-1 FW)
b
a
c
c
Col-0
acr2-2
hac1-1
acr2-2 hac1-1

continue

Figure S5. *HAC1* and *ACR2* do not interact additively as part of the metabolism of arsenic. Wild-type Col-0, single *acr2-2* and *hac1-1* mutants and the *acr2-2 hac1-1* double mutant were grown hydroponically for three weeks and 5 μM arsenate added for analysis thereafter. Accumulation of arsenate and arsenite was monitored in both roots (**A**) and shoots (**B**) for all genotypes. The uptake of arsenate (**C**) and efflux of arsenite (**D**) was also monitored and calculated from changes in their concentrations in the hydroponic growth media. Letters above bars indicate statistically different groups using a one way ANOVA followed by least significant difference (LSD) test at the probability of $p<0.05$. Data represent means ± S.E. (n = 4). Raw data available in Data S9.
