# Supplementary material for: Genome-wide Association Mapping Identifies a New Arsenate Reductase Enzyme Critical for Limiting Arsenic Accumulation in Plants
Source: PLoS Biol. 2014 Dec 2;12(12):e1002009. doi: 10.1371/journal.pbio.1002009 (PMC4251824; doi:10.1371/journal.pbio.1002009)
Supplement: Figure S6 — Loss-of-function of HAC1 does not affect accumulation of phosphorus in shoots. The concentration of total phosphorus was quantified by ICP-MS in leaves of wild-type Col-0, Kr-0, and hac1-1 and hac1-2 plants grown in artificial soil for 5 wk. Letters above bars indicate statistically different groups using a one-way ANOVA followed by least significant difference (LSD) test at the probability of p<0.05. Data represent means ± S.E. (n = 12). Raw data available in Data S13. (PDF) [file pbio.1002009.s006.pdf]

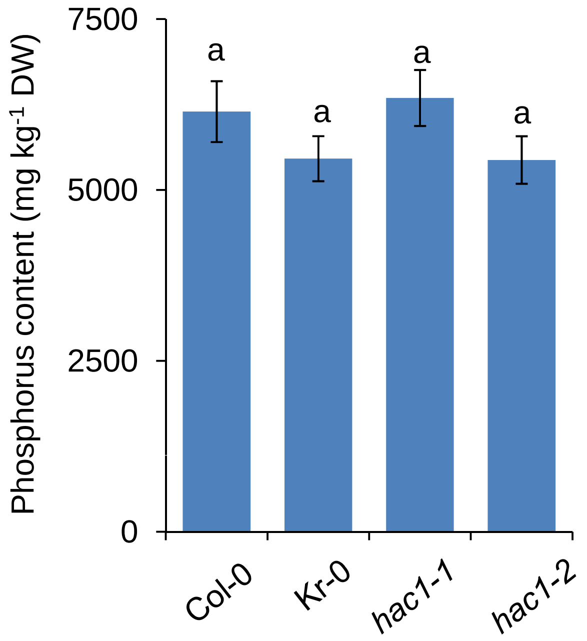

Figure S6. Loss-of-function of *HAC1* does not affect accumulation of phosphorus in shoots. The concentration of total phosphorus was quantified by ICP-MS in leaves of wild-type Col-0, Kr-0, and *hac1-1* and *hac1-2* plants grown in artificial soil for 5-weeks. Letters above bars indicate statistically different groups using a one way ANOVA followed by least significant difference (LSD) test at the probability of $p<0.05$. Data represent means ± S.E. (n = 12). Raw data available in Data S13.
