# Supplementary material for: Genome-wide Association Mapping Identifies a New Arsenate Reductase Enzyme Critical for Limiting Arsenic Accumulation in Plants
Source: PLoS Biol. 2014 Dec 2;12(12):e1002009. doi: 10.1371/journal.pbio.1002009 (PMC4251824; doi:10.1371/journal.pbio.1002009)
Supplement: Figure S7 — HAC1 and ACR2 sequence similarity. DNA Sequence similarity between A. thaliana HAC1 and the 207 nucleotide sequence in the 3′ UTR of ACR2 used for RNA interference in a previous study [23]. Numeric positions of nucleotides indicated above reference to the starting position of the ACR2-RNAi fragment [23], and below the sequences the numbers reference to the start codon for A. thaliana HAC1. (PDF) [file pbio.1002009.s007.pdf]

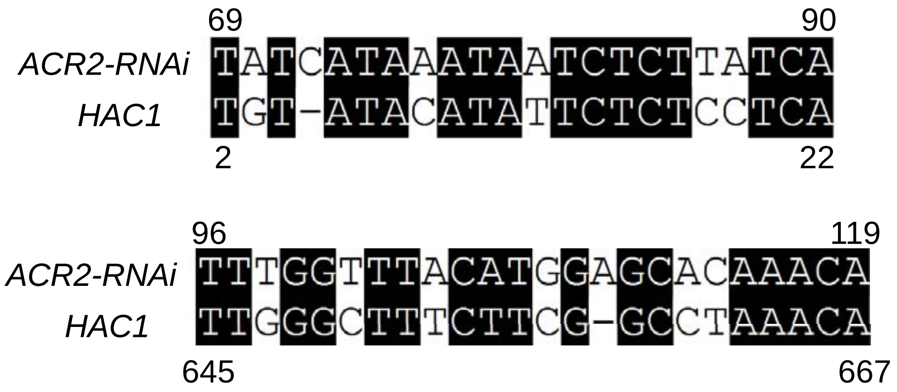

Supplementary Figure 7. *HAC1* and *ACR2* sequence similarity. DNA Sequence similarity between *A. thaliana HAC1* and the 207 nucleotide sequence in the 3’ UTR of *ACR2* used for RNA interference in a previous study (23). Numeric positions of nucleotides indicated above reference to the starting position of the *ACR2-RNAi* fragment (23), and below the sequences the numbers reference to the start codon for *A. thaliana HAC1*.
